# CADLIVE optimizer: web-based parameter estimation for dynamic models

**DOI:** 10.1186/1751-0473-7-9

**Published:** 2012-08-28

**Authors:** Kentaro Inoue, Kazuhiro Maeda, Yuki Kato, Shinpei Tonami, Shogo Takagi, Hiroyuki Kurata

**Affiliations:** 1 Department of Bioscience and Bioinformatics, Kyushu Institute of Technology, Iizuka, Fukuoka, 820-8502, Japan

**Keywords:** Simulator, Optimization, Genetic algorithm, Dynamic model

## Abstract

Computer simulation has been an important technique to capture the dynamics of biochemical networks. In most networks, however, few kinetic parameters have been measured in vivo because of experimental complexity. We develop a kinetic parameter estimation system, named the CADLIVE Optimizer, which comprises genetic algorithms-based solvers with a graphical user interface. This optimizer is integrated into the CADLIVE Dynamic Simulator to attain efficient simulation for dynamic models.

## Introduction

The simulation of dynamic models is a powerful approach that can be used for: (i) checking the consistency of a postulated model with a set of experimental measurements, (ii) answering ‘what if?’ questions and (iii) exploring possible behaviors of a model [1]. Differential equations play a critical role in the dynamic simulation of biochemical network models and the robustness analysis of them.

A major problem for the dynamic modeling is to know the values of kinetic parameters in vivo, but it is very hard to measure the exact values of them due to experimental complexity. Stochastic methods including evolutionary (genetic) algorithms can provide high-quality solutions in less computational cost [2,3]. At present evolutionary searches are widely used to optimize a dynamic model of biochemical networks (SBML-PET [4], libSRES [5], BioNessie [6], and AMIGO [7]).

**Figure 1:**
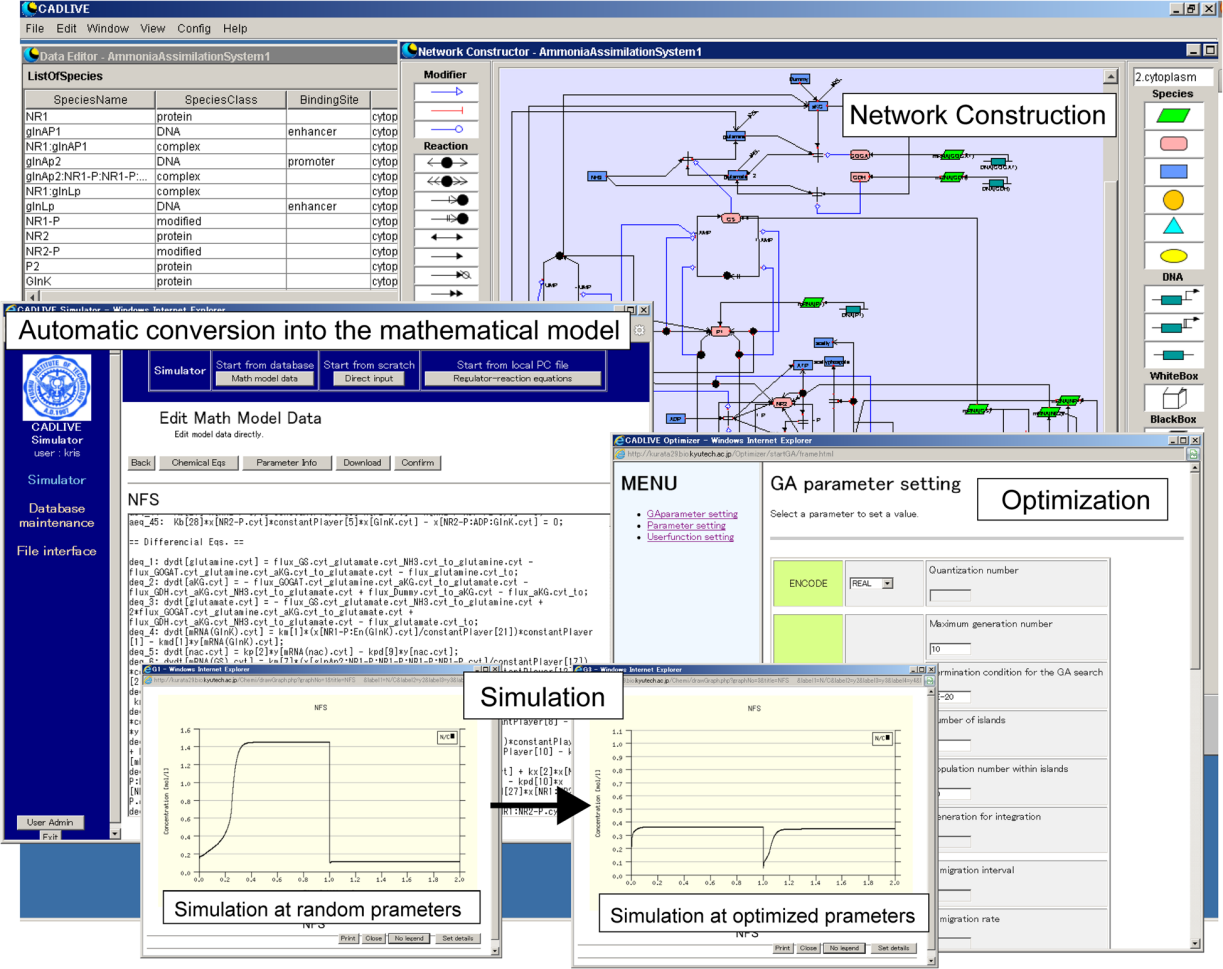
The CADLIVE Optimizer integrated into the CADLIVE Dynamic Simulator.

We presented a web-based dynamic simulator, the CADLIVE (Computer-Aided Design of LIVing systEms) Dynamic Simulator that takes a strong advantage in automatic conversion from a biochemical network map to its associated dynamic model [8-11], while it has not implemented any built-in parameter estimator yet. Users are required to manually edit computer programs and to handle complicated procedures on the Linux command line, hampering the optimization in the CADLIVE system. To overcome these problems, we develop an optimization module with a user-friendly Graphical User Interface (GUI) and integrate it into the CADLIVE Dynamic Simulator (Figure 1).

## Methods

### Optimizer

Genetic algorithms (GAs) are known as one of the algorithms that can seek out the global minimum, based on the heuristic assumptions that the best solutions will be found in the regions of the parameter space containing a relatively high proportion of good solutions and that these regions can be explored by the genetic operators of selection, crossover, and mutation. In 2005 we developed the GA-based optimization programs for the CADLIVE Dynamic Simulator [9]. Details of description for GAs are in Additional file 1. However, these programs are provided in the C language form, thus users have to manually edit several functions and parameters necessary for a search by GAs and use the LINUX command lines. This manual operation requires users to learn the skills and knowledge of programming and evolutionary search, which reduces the efficiency of the parameter estimation process. In this report, we develop the GUI application that enables users to intelligibly handle the optimization programs without these annoying procedures.

### Parameter and function settings

**Figure 2:**
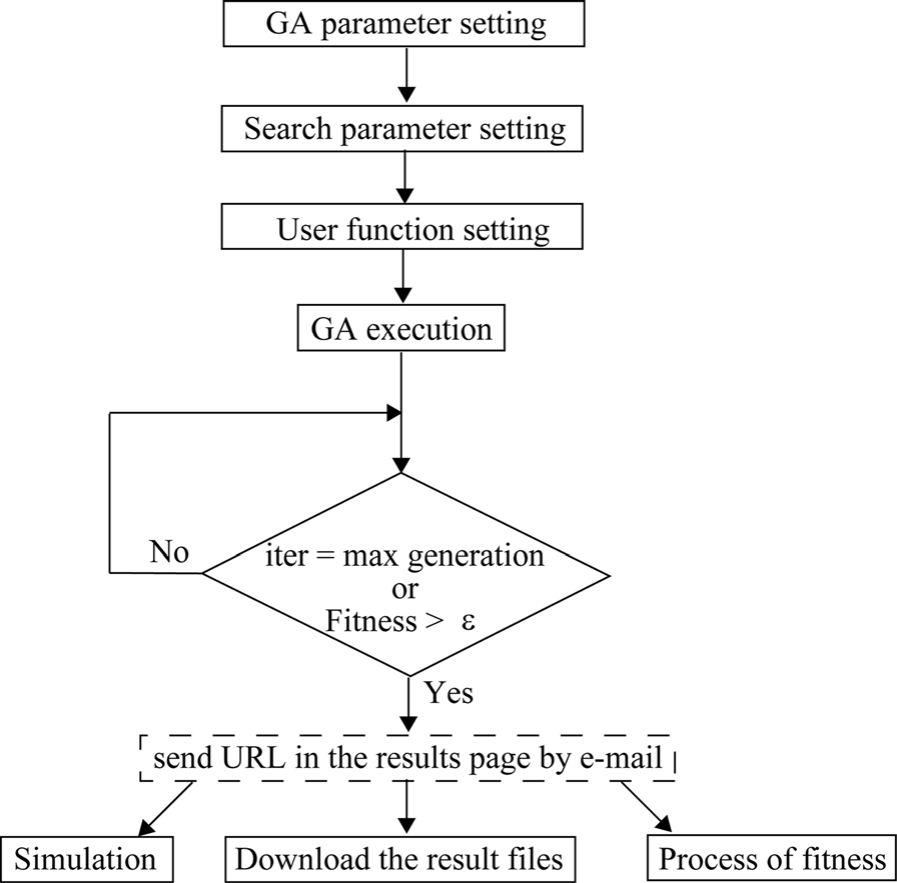
**A flow chart for performing the CADLIVE Optimizer.** Users do the first three settings. A search by GAs finishes when the number of iterations is the max generation or the fitness value is more than ϵ. ϵ is very small, e.g. -10^-20^. After optimization, users can obtain three functions: simulation with the optimized parameters, download the result files (containing all the employed parameters and simulated data), and process of fitness (a change in the fitness value with respect to generation).

Input and output files are defined to control the search programs as the application modules independent of mathematical equations, so that users just write the input files to start a GA search without editing any search algorithms. Three input files: the GA parameter setting file, the search parameter setting file, and the user function setting file, are made in the server and defined for optimization for a dynamic model (Figure 2), as follows.

#### GA parameter setting file

Users set the parameters necessary for GAs, i.e. encode method, GA type, digenesis, immigration, crossover, and mutation.

#### *Search parameter setting file*

It is derived from the parameter files generated by the CADLIVE Dynamic Simulator. Users select search parameters and edit their initial values and search ranges.

#### User function setting file

It is derived from the usrfunc files generated by the CADLIVE Dynamic Simulator. Users make the objective function necessary for optimization. The usrfunc file has two contents: the mathematical equations and objective function. The mathematical equations are provided by the CADLIVE Dynamic Simulator. In objective function setting, users can select the sum of squared errors (SSE) by default, where users upload experimental or reference time-course data necessary for the calculation of SSE. If necessary, users can arbitrarily edit the objective function according to the grammar of the C language. Simulated data are interpolated using the spline interpolation function.

### GA execution

After setting the input files using the GUI, users start a GA search. When the optimization finishes, users obtain the three functions: "Simulation", "Process of fitness", and "Download", as follows.

#### Simulation

Users can simulate the optimized dynamic model with respect to the objective function.

#### Process of fitness

This function displays the changes in the fitness values with respect to generation. The fitness value shows how much the simulated data agree to the requirement given by the objective function.

#### Download the result files

Users can download three input files for the GA parameter setting, search parameter setting, and user function setting and the two output files for the GA result and optimized search parameters.

### Implementation

A personal computer (CPU:Intel Xeon E3110 3 GHz, RAM:4GByte) is used as the server machine. The GUI program is written in PHP 4.4.7 on the LINUX Cent OS 5.3 and integrated into the CADLIVE Dynamic Simulator. The C compiler on LINUX processes the three input files: the GA parameter setting file, search parameter setting file, and user function setting file, to execute the search by GAs. The progress of the GA search is supervised by cron in Linux. The mail service is implemented that notifies the completion of optimization. The simulated results with the optimized parameters are drawn by the library of the CADLIVE Dynamic Simulator. JpGraph (http://www.jpgraph.net) is used to display the process of fitness values. The manuals of the CADLIVE Optimizer are provided in Additional file 2.

## Results and discussion

We automatically converted three example models: a simple enzyme reaction model, an *E. coli* heat shock response system [12], and an *E. coli* nitrogen assimilation system [13] (see Additional file 3, Additional file 4) into their associated dynamic models by using the CADLIVE Dynamic Simulator, and subsequently estimated the kinetic parameter values of them by the CADLIVE Optimizer. An enzyme reaction model is the simplest model to let users understand the basic functions of the optimizer. The heat shock response system is used to tell how ordinary biochemical models are optimized, where the SSE between the experimental and simulated time course data are employed as the default objective function. The nitrogen assimilation system shows that an arbitrary objective function can be edited using the experimental data [13]. The proposed GUI application greatly facilitates the feasibility of the optimization programs, by removing complicated procedures such as the edition of the files necessary for setting GAs and objective functions and handling of the LINUX commands.

CADLIVE has originally been developed to perform rational computer-aided design of biochemical networks and used for various studies [12,13]. In this report, as an extension of CADLIVE, we propose the CADLIVE Optimizer, a powerful tool for optimizing a mathematical model generated by the CADLIVE Dynamic Simulator. The CADLIVE Optimizer attains a critical progress for developing the standard technology for automatic optimization of dynamic models, i.e., the automatic generation of a dynamic model with tuned kinetic parameters without any complicated operations.

## Competing interests

The authors declare that they have no competing interests.

## Authors’ contributions

KI, YK, ST and ST wrote source code for Optimizer. KM provided biological models. KI and HK wrote the paper. All authors read and approved the final manuscript.

## Supplementary Material

Additional file 1 Description of GAs employed in the CADLIVE Optimizer. Click here for file

Additional file 2 Instruction for the CADLIVE Optimizer. Click here for file

Additional file 3 Description of three example models. Click here for file

Additional file 4 Files for the example models. Click here for file
