# Supplementary material for: CADLIVE optimizer: web-based parameter estimation for dynamic models
Source: Source Code Biol Med. 2012 Aug 28;7:9. doi: 10.1186/1751-0473-7-9 (PMC3457844; doi:10.1186/1751-0473-7-9)
Supplement: Additional file 1 — Description of GAs employed in the CADLIVE Optimizer. [file 1751-0473-7-9-S1.doc]

**Additional file 1. Description for GAs employed in the CADLIVE Optimizer**

## 1. Encode method

This optimization program presents the real-coded GA (RGA) and the bit-string GA (BGA) as the method that encodes design variables. For the bit-string GAs, the binary coding or gray coding is employed as the method that exchanges between integers and bit-string (0,1).

### 1-1. Bit-string GA

The bit-string GA encodes the design variables as the bit string that consists of 0 and 1. The bit-string GA divides the range of variables into $2^{n}$ regions, and assigns the integer of $0,\ldots,2^{n}-1$ for each region in a numerical order, thereby converting the integer to the bit-string. Two encoding methods as follows:

- Binary coding: converting an integer by binary arithmetic
- Gray coding: converting an integer with a binary code under the condition that the Haming distance between two codes to continue is always kept 1. The Haming distance is defined as the number of the figures with different values.

Table Binary and gray codes

| Integer | Binary code | Gray code |
| --- | --- | --- |
| 0 | 000 | 000 |
| 1 | 001 | 001 |
| 2 | 010 | 010 |
| 3 | 011 | 011 |
| 4 | 100 | 110 |
| 5 | 101 | 111 |
| 6 | 110 | 101 |
| 7 | 111 | 100 |

### 1-2. Real GA

The real GAs employ the sequence of real values as the chromosome.

## 2. GA type

This module presents two types of GAs, the distributed GA (DGA, island model) and the distributed and integrated GA (DIGA).

### 2-1. Distributed GA

The distributed GA divides the population into multiple islands. The individuals of each island evolve within the island, and some individuals immigrate mutually among the islands. The immigration method can be selected from random swap, random ring, and no immigration.

- Random swap: selecting immigrants randomly out of randomly selected islands under the given immigration rate in order to exchange two individuals between the islands. The elites of each island are not allowed to immigrate.
- Random ring: determining the destination of immigrants so that the way between the destination and origin of the immigrants forms a ring. The elites of each island are not allowed to immigrate.

### 2-2. Distributed and integrated GA (DIGA)

The DIGA divides the population into multiple islands as well as DGA. Each island evolves independently until all the islands are integrated to a new island at the generation number for integration. At their integration, the elites of each island are gathered to the new one, and the remaining individuals are selected randomly. The DIGA is not allowed to operate the immigration.

## 3. Generation alternation

The GA module presents two methods for generation alternation, the ordinary generation alternation method (employing elite conservation method), and Minimal Generation Gap (MGG). The ordinary method is sometimes called the simple GA, but such a name may cause users to misunderstand the methods. Here, we name the ordinary generation alternation method.

### 3-1. Ordinary generation alternation method

The ordinary method replaces all the parents by children. Actually, the parents except the elites are replaced, because the elite conservation strategy is employed. If users want to replace all the parents including the elites, set “the number of elites” as zero.

The roulette, tournament, and random selections are presented as the methods for selecting the parents to crossover. The roulette is made based on the difference:

(difference) = (fitness of an individual) – (the lowest fitness of the island).

### 3-2. Minimal Generation Gap (MGG)

The MGG is the most desirable model that can avoid early convergence and suppress evolutionary stagnation. The procedure of MGG can be summarized in the following box.

1. Create an initial population randomly

2. Sample two individuals randomly without replacement from the population

3. Generate children from the selected parents and characterize them

4. Replace two individuals (parents) by the best individual and the roulette-selected individual, resulting in the next generation.

The above method is the original MGG. Some crossover methods require more than two parents. Thus, the crossover is defined by the rules:

- BLX-α, $n$-point mutation: The parents to replace are the same ones to crossover.
- UNDX, UNDX-m, SPX: Two parents to replace are selected randomly out of all the parents that have been employed to generate children.

## 4. Crossover method

The crossover method to apply depends on the encoding methods, the bit-string GA (BGA) and the real GA (RGA). In this section, [RGA] indicates that the crossover is available for RGA, [BGA] for BGA.

### 4-1. BLX-α [RGA]

The BLX is a simple algorithm among the real-coded GAs, but its performance is not guaranteed in the variable-dependent functions.

$\begin{matrix} c_{i}=u(\min(p_{i}^{1},p_{i}^{2})-\alpha I_{i},\max(p_{i}^{1},p_{i}^{2})+\alpha I) \\ I_{i}=|p_{i}^{1}-p_{i}^{2}| \end{matrix}$,

where $\mathbf{p}^{1}=(p_{1}^{1},\ldots,p_{n}^{1}),\mathbf{p}^{2}=(p_{1}^{2},\ldots,p_{n}^{2})$ are the parents, $\mathbf{c}=(c_{1},\ldots,c_{n})$is the child, and $u(\cdot)$ represents the function that generates a uniform random number within the closed region of the parentheses.

### 4-2. UNDX / UNDX-m [RGA]

The UNDX-m has been proposed to improve a search performance on an optimization problem with poorly scale coordinate systems. The UNDX-m generates offspring vector values by sampling values from the m dimensional space that the m+1 parents span around their middle point. The UNDX is the same as the UNDX-m at m = 1 except some respects. The prototype algorithm of the UNDX-m in the n-dimensional parameter space is provided by:

(1) Select m +1 parents $\mathbf{x}^{1},\ldots,\mathbf{x}^{m+1}$randomly from the population.

(2) Let the center of mass of these parents

$\mathbf{p}=\frac{1}{m+1}\sum_{i=1}^{m+1} \mathbf{x}^{i}$,

and let the difference vector between $\mathbf{x}^{i}$and $\mathbf{p}$be $\mathbf{d}^{i}=\mathbf{x}^{i}-\mathbf{p}$.

(3) Select another parent $\mathbf{x}^{m+2}$ from the population randomly

(4) Let *D* be the length of the component of $\mathbf{d}^{m+2}=\mathbf{x}^{m+2}-\mathbf{p}$orthogonal to $\mathbf{d}^{1},\ldots,\mathbf{d}^{m}$.

(5) Let $\mathbf{e}^{1},\ldots,\mathbf{e}^{n-m}$ be orthogonal bases of the subspace orthogonal to the vectors $\mathbf{d}^{1},\ldots,\mathbf{d}^{m}$.

(6) Generate the child $\mathbf{x}^{c}$by:

$\mathbf{x}^{c}=\mathbf{p}+\sum_{i=1}^{m} w_{i}\mathbf{d}^{i}+\sum_{i=1}^{n-m} v_{i}D\mathbf{e}^{i}$,

where$w_{i},v_{i}$are the random numbers that follow normal distributions $N(0,\sigma_{\xi}^{2}),N(0,\sigma_{\eta}^{2})$, respectively. The parameters, $\sigma_{\xi}$and $\sigma_{\eta}$, are provided by:

$$\sigma_{\xi}=\alpha/\sqrt{m},\begin{matrix} & \end{matrix}\sigma_{\eta}=\frac{1}{\sqrt{n-m}}\frac{\sqrt{m+1}}{\sqrt{m+2}}\frac{\sqrt{3}}{\sqrt{2}}\beta$$

where α= 1.0 and β= 0.35 are recommended.

The UNDX is carried out as follows:

(1) Select three parents $\mathbf{x}^{1},\mathbf{x}^{2},\mathbf{x}^{3}$ randomly.

(2) Let the center of mass of these parents:

$\mathbf{x}^{p}=(\mathbf{x}^{1}+\mathbf{x}^{2})/2$,

and let the difference vector $\mathbf{d}=\mathbf{x}^{1}-\mathbf{x}^{2}$.

(3) Let *D* to be the distance between the third parent$\mathbf{x}^{3}$and the line connecting **x**^1^ to **x**^2^.

(4) Generate a child **x**^c^ by the following equation:

$\mathbf{x}^{c}=\mathbf{x}^{p}+\xi\mathbf{d}+D\sum_{i=1}^{n-1} \eta_{i}\mathbf{e}_{i}$,

where $\xi$ and $\eta_{i}$ are the random numbers that follow normal distributions $N(0,\sigma_{\xi}^{2})$and $N(0,\sigma_{\eta}^{2})$, respectively. The parameters, $\sigma_{\xi}$ and $\sigma_{\eta}$, are given by:

$$\sigma_{\xi}=\alpha,\begin{matrix} & \end{matrix}\sigma_{\eta}=\beta/\sqrt{n}$$

where α = 0.5 and β= 0.35 are recommended.

Difference between UNDX and UNDX-m

The recommended value of α is a half of that of the UNDX-m, because the UNDX employs the difference vector between the parents instead of the difference vector based on the center of mass. Actually, both are the same. On the other hand, for the sub-search component, the UNDX-m provides:

$\sigma_{\eta}=\beta/\sqrt{n-1}$ at m = 1,

where the difference is indicated clearly between them.

Table 1 Difference between UNDX and UNDX-m

|  |  | Revision on sub-search component |
| --- | --- | --- |
| UNDX | $\sigma_{\xi}=\alpha$ | $\sigma_{\eta}=\beta/\sqrt{n}$ |
| UNDX-m | $\sigma_{\xi}=\alpha/\sqrt{m}$ | $\sigma_{\eta}=\frac{1}{\sqrt{n-m}}\frac{\sqrt{m+1}}{\sqrt{m+2}}\frac{\sqrt{3}}{\sqrt{2}}\begin{matrix} & \end{matrix}\beta(m=1,\beta/\sqrt{n-1})$ |

Note: Rank cancellation

In the UNDX-m, the space that the difference vectors span is the main search component, and the orthogonal subspaces are the minor search components. If the difference vector sets that the main search component belongs to have any dependent element, the dimension of the main search space is less than the number of the vectors that span it, canceling the rank.

When the rank is cancelled, the optimization module does not change the main search component, and lets the minor search component to be the subspace orthogonal to the space (P) that independent vectors of the mains search components span. The parameter *D* is set as the component of the vector $\mathbf{d}^{m+2}$ orthogonal to the space (P).

### 4-3. SPX [RGA]

SPX generates offspring vector values by uniformly sampling values from the simplex formed by multiple parent vectors.

(1) Seletct m+1 parents randomly from the population.

(2) Let the center of mass of the parents $\vec{G}$ be

.

(3) The vector are determined as follows:

,

where ε is the extension coefficient (ε > 0), $r_{k}$ is provided by random uniform function:

$\begin{matrix} r_{k}=(u(0,1))^{1/(k+1)} & (k=0,\ldots,n-1) \end{matrix}$.

The coefficient ε is provided by:

$$\varepsilon=\sqrt{n+2}$$

(4) The child is provided by

### 4-4. N point crossover [BGA]

N points to crossover are selected at a chromosome randomly, and the parameters between the crossing points are replaced alternately by the corresponding parameters of a parental chromosome.

## 5. Mutation method

The mutation methods to apply depend on BGA or RGA. In this section, the mutation method for RGA is explained. The individuals that have been selected out of an island at a given mutation rate are mutated according to the various methods, where $(x_{1},\ldots,x_{n})$ is the coordinate of an individual, and $(z_{1},\ldots,z_{n})$ is the coordinate that shows the best individual in all of the individuals within the island.

### 5-1. Uniform mutation within the region [RGA]

The vector of the selected individual is changed under the uniform random distribution within the given region.

### 5-2. Uniform mutation with fixed width [RGA]

A mutated vector is given by the function:

$u(x_{i}-w,x_{i}+w)$,

which generates a uniform random number between $x_{i}-w$and $x_{i}+w$, where *w* is the width that users give arbitrarily.

### 5-3. Normal mutation with fixed width [RGA]

A mutated vector is provided by the function:

$u(x_{i}-w,x_{i}+w)$,

which generates a normal random number between $x_{i}-w$and$x_{i}+w$, where *w* is the width that users give arbitrarily. The coordinate of the mutated vector follows a normal distribution:

$N(x_{i},s^{2})$ ,

where *s* is the standard deviation that users can give arbitrarily.

### 5-4. Uniform mutation with variable width [RGA]

A mutated vector is provided by the function:

$u(x_{i}-w_{v},x_{i}+w_{v})$,

which generates a uniform random number between $x_{i}-w_{v}$ and $x_{i}+w_{v}$, where $w_{v}$ is given by:

$w_{v}=|z_{i}-x_{i}|$.

### 5-5. Normal mutation with variable width [RGA]

A mutated vector is provided by the function:

$u(x_{i}-w,x_{i}+w)$,

which generates a normal random number between $x_{i}-w$ and $x_{i}+w$, where *w* is the width that users give arbitrarily. The coordinate of the generated vector follows a normal distribution:

$N(x_{i},s_{v}^{2})$ ,

where the standard deviation is $s_{v}=|z_{i}-x_{i}|$.

### 5-6. Bit reverse mutation [BGA]

A vector is mutated by reversing the bit according to the given mutation rate.
