# Supplementary material for: CADLIVE optimizer: web-based parameter estimation for dynamic models
Source: Source Code Biol Med. 2012 Aug 28;7:9. doi: 10.1186/1751-0473-7-9 (PMC3457844; doi:10.1186/1751-0473-7-9)
Supplement: Additional file 2 — Instruction for the CADLIVE Optimizer. [file 1751-0473-7-9-S2.doc]

**Additional file 2. Instruction of the CADLIVE Optimizer**

[1. Overview 1](#__RefHeading___Toc318365849)

[2. Network map generated by the CADLIVE GUI Network Constructor 2](#__RefHeading___Toc318365850)

[3. Conversion of a network map into the mathematical model by the CADLIVE Dynamic Simulator 4](#__RefHeading___Toc318365851)

[4. Optimization of the kinetic parameters of the equations by the CADLIVE Optimizer 9](#__RefHeading___Toc318365852)

[4-1. GA parameter setting 9](#__RefHeading___Toc318365853)

[4-2. Search parameter setting 13](#__RefHeading___Toc318365854)

[4-3. User function setting 15](#__RefHeading___Toc318365855)

[4-4. GA execution 17](#__RefHeading___Toc318365856)

[4-5. Confirmation of the results 19](#__RefHeading___Toc318365857)

[4-5-1. Simulation 20](#__RefHeading___Toc318365858)

[4-5-2. Download the result files 21](#__RefHeading___Toc318365859)

[4-5-3. Process of fitness 22](#__RefHeading___Toc318365860)

[5. References 23](#__RefHeading___Toc318365861)

Recommended web browser: Internet Explorer

URL: http://kurata23.bio.kyutech.ac.jp/Life/index.html

# 1. Overview

There are three steps for optimizing a mathematical model by CADLIVE (**Fig.1**). First, a biological network is built by the CADLIVE Network Constructor . Second, the biological network is automatically converted into its associated mathematical model by the CADLIVE Dynamic Simulator . Finally, the CADLIVE Optimizer estimates the values of kinetic parameters using the objective function built based on experimental data. Here, we illustrate the dynamic simulation of a heat shock response system.


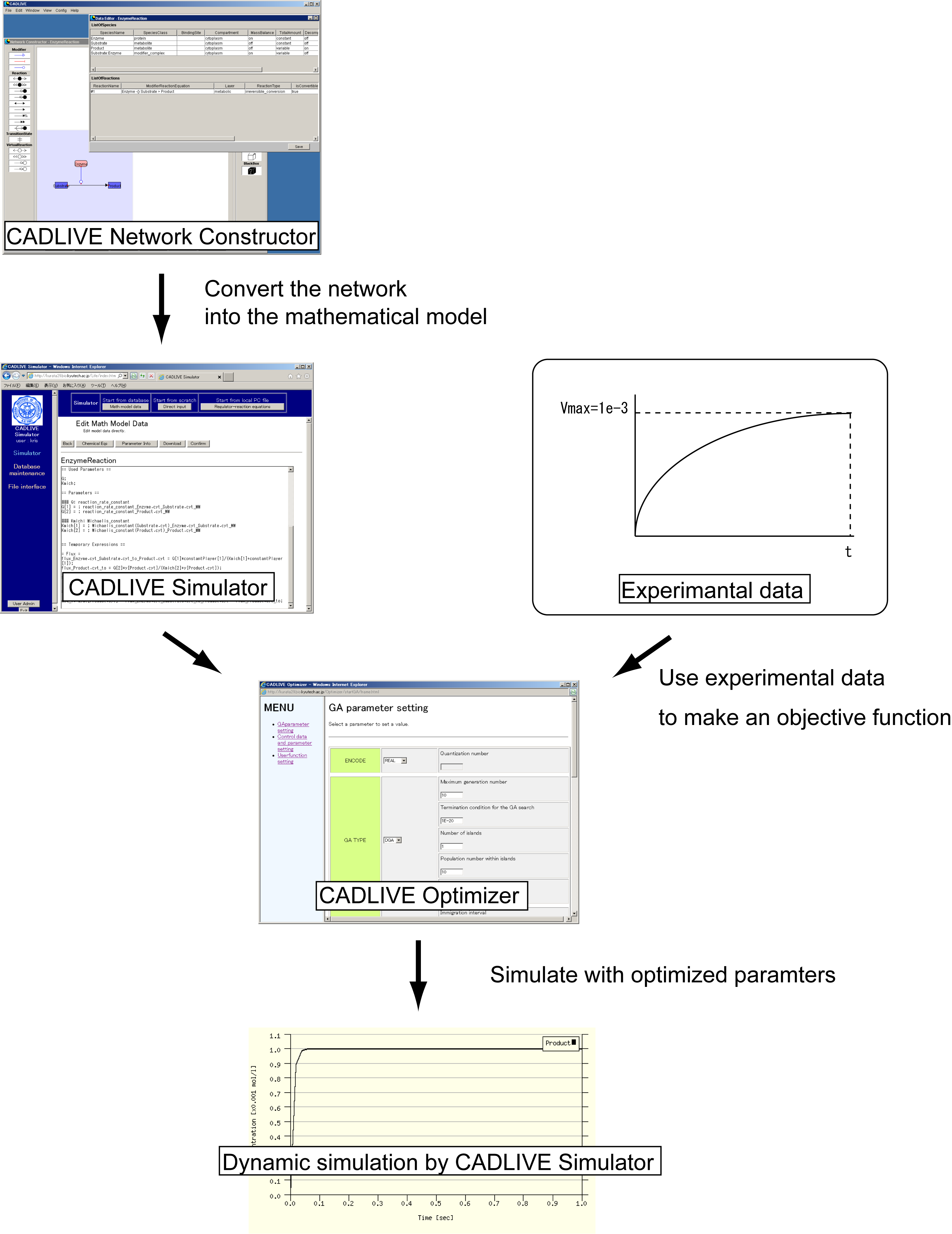


Fig.1 Flow in CADLIVE Optimizer

# 2. Network map generated by the CADLIVE GUI Network Constructor

The CADLIVE GUI Network Constructor is a software suite for drawing a large-scale map of molecular interactions and for registering their associated regulator-reaction equations (RREs) in an extension of SBML level 2. A biochemical network map is drawn with the symbols of molecules (right) and reactions (left) by the CADLIVE GUI Network Constructor. The biochemical network map is automatically converted into the SBML-based regulator reaction equations. The heat shock response system is drawn as shown **Fig. 2**. Then, in the window of Data Editor (**Fig.3**), users set the parameters necessary for dynamic simulation. The instruction manual of the CADLIVE GUI Network Constructor can be downloaded from http://www.cadlive.jp.


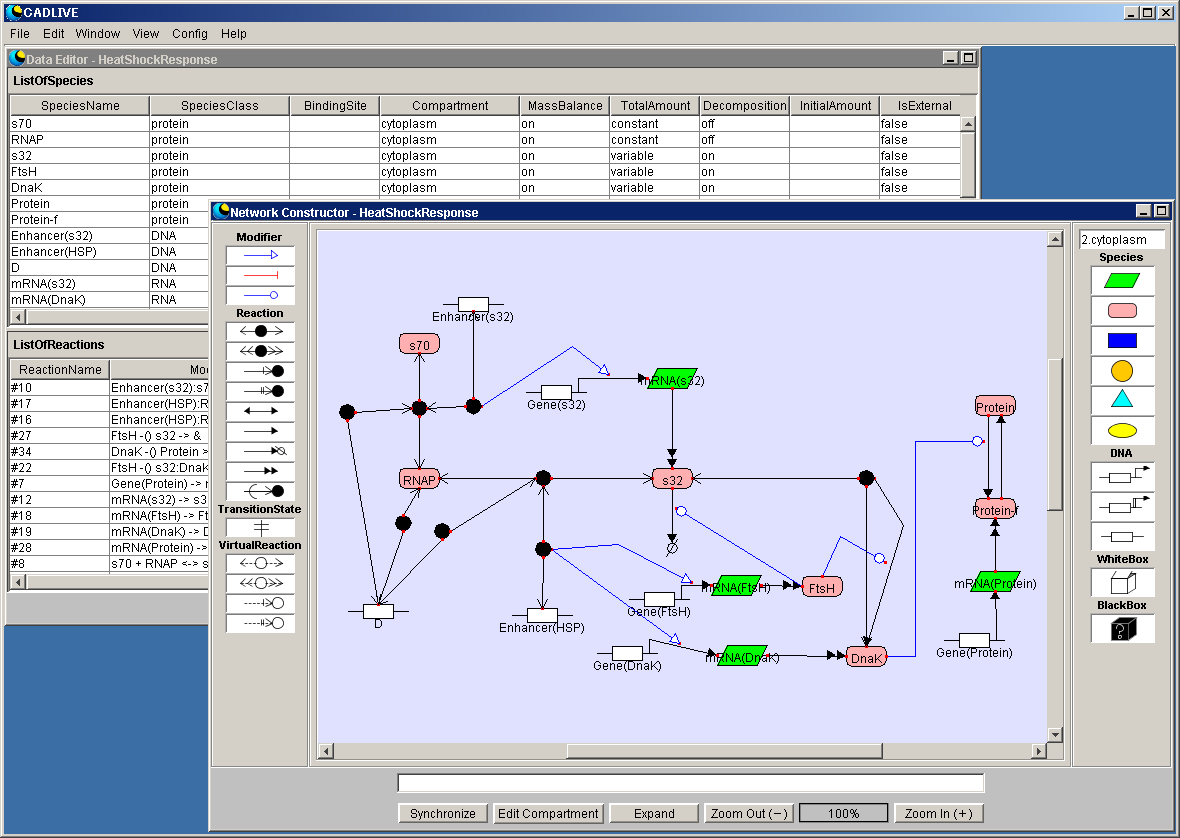


Fig.2 The heat shock response system by the CADLIVE GUI Network Constructor


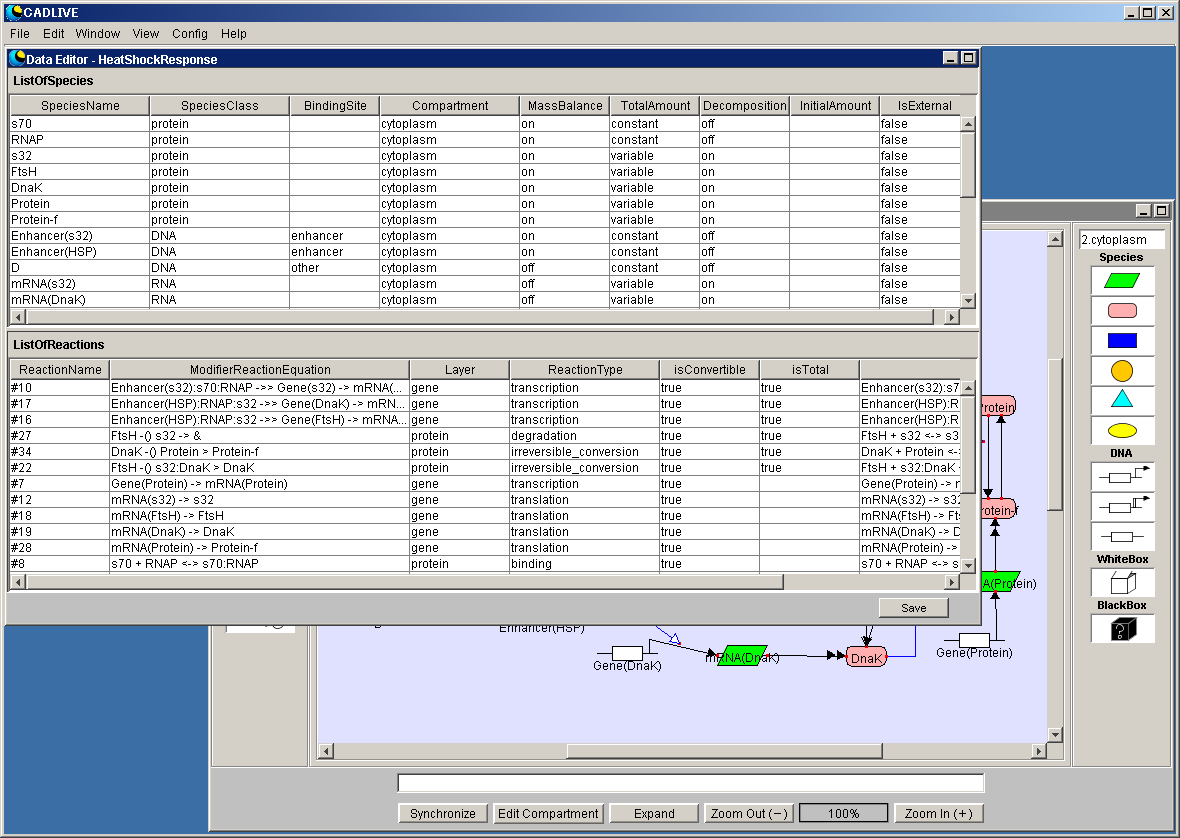


Fig.3 The window of the text data editor

# 3. Conversion of a network map into the mathematical model by the CADLIVE Dynamic Simulator

The CADLIVE Dynamic Simulator provides a rule-based automatic way to convert biochemical network maps into mathematical models, which enables simulating their dynamics without going through all of the reactions down to the details of exact kinetic parameters. Users select the type of mathematical models: ordinary transcription and translation equations (TT), simplified Michaelis-Menten equations (MM), Two-Phase Partition (TPP) model, Conventional Mass Action (CMA), and General Mass Action (GMA) (**Fig.8**), and the analysis type: dynamic and steady-state (**Fig.10**). In control data for simulation, users select the solver type: the Runge-Kutta method, the step-adaptive Runge-Kutta method, and the Numerical Differentiation Formulas (NDF) (**Fig.11**), time span, and time step-size (**Fig.11**). Users can set the parameters necessary for the Newton-Raphson method (**Fig.11**). Then users put the values of kinetic parameters and the initial values (**Fig.12**). The instruction manual of the CADLIVE Dynamic Simulator can be freely downloaded from http://www.cadlive.jp.

Users input users’ name and password.

User name: cadlive

Password: simulator


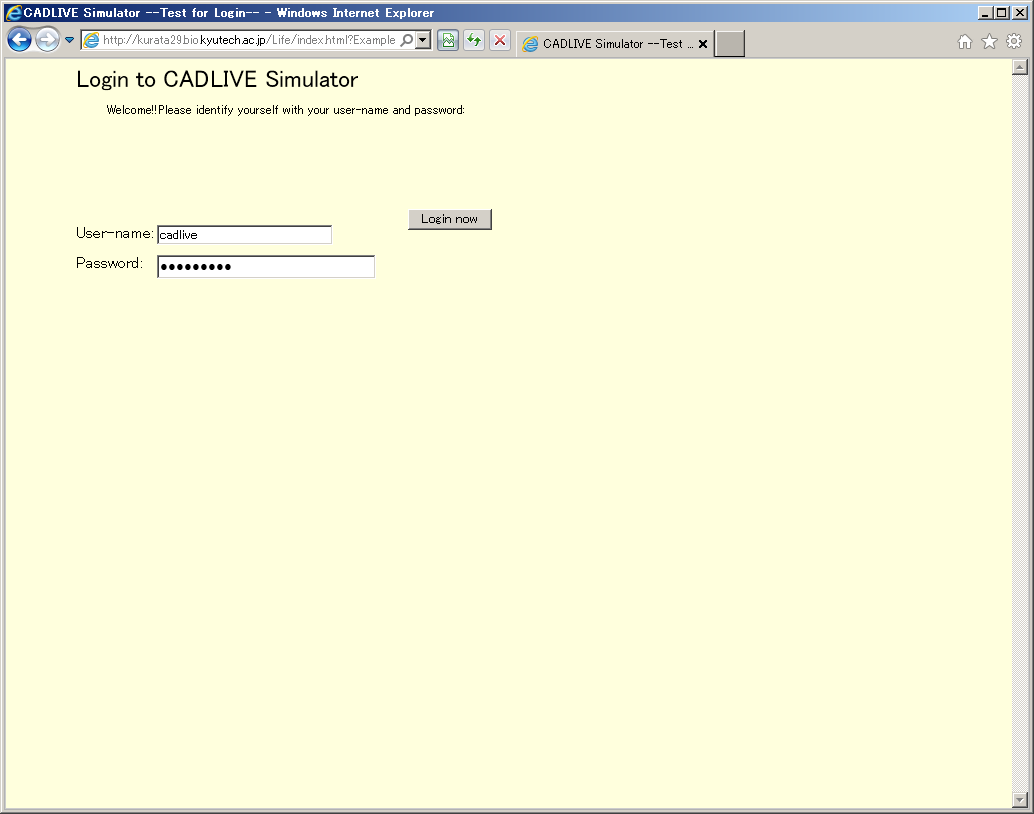


Fig.5 CADLIVE Dynamic Simulator login screen

Users click the “Simulator” on the left side.


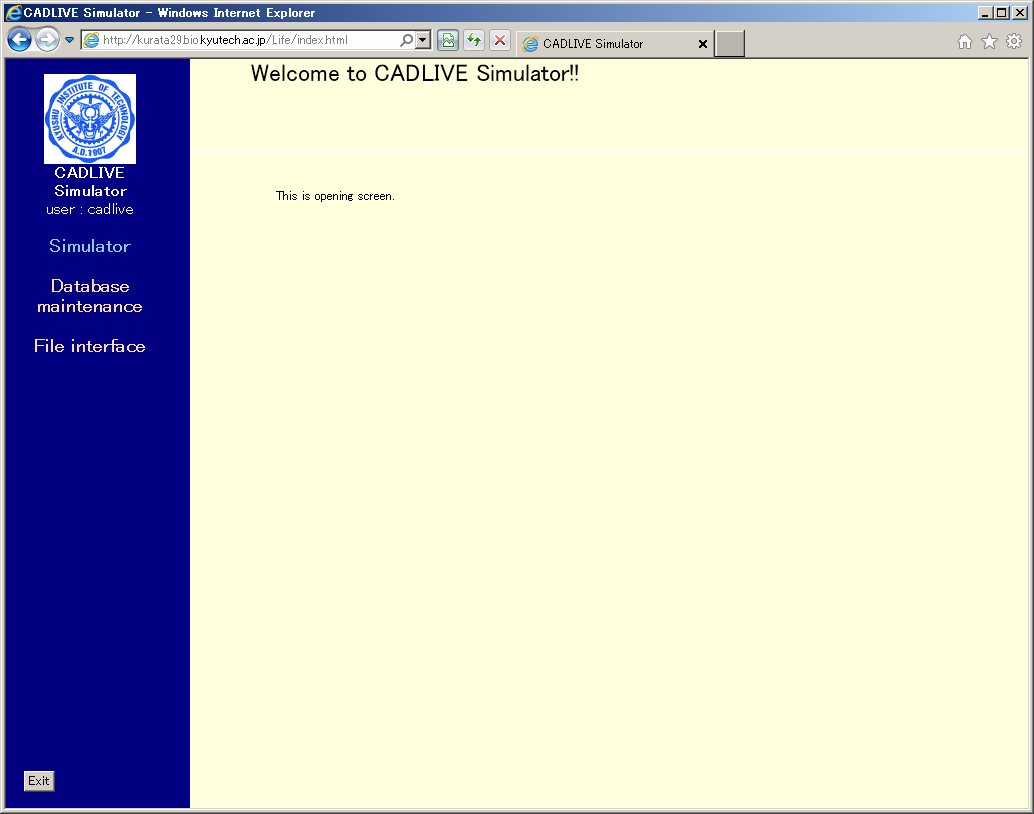


Fig.6 CADLIVE Dynamic Simulator start screen

Users click the “Regulator-reaction equations” button on the top side. Then, users upload a CADLIVE format file in users’ PC. Here, upload the file “HeatShockResponse.XML” from the folder “HeatShockResponse” in **Additional file 3**.


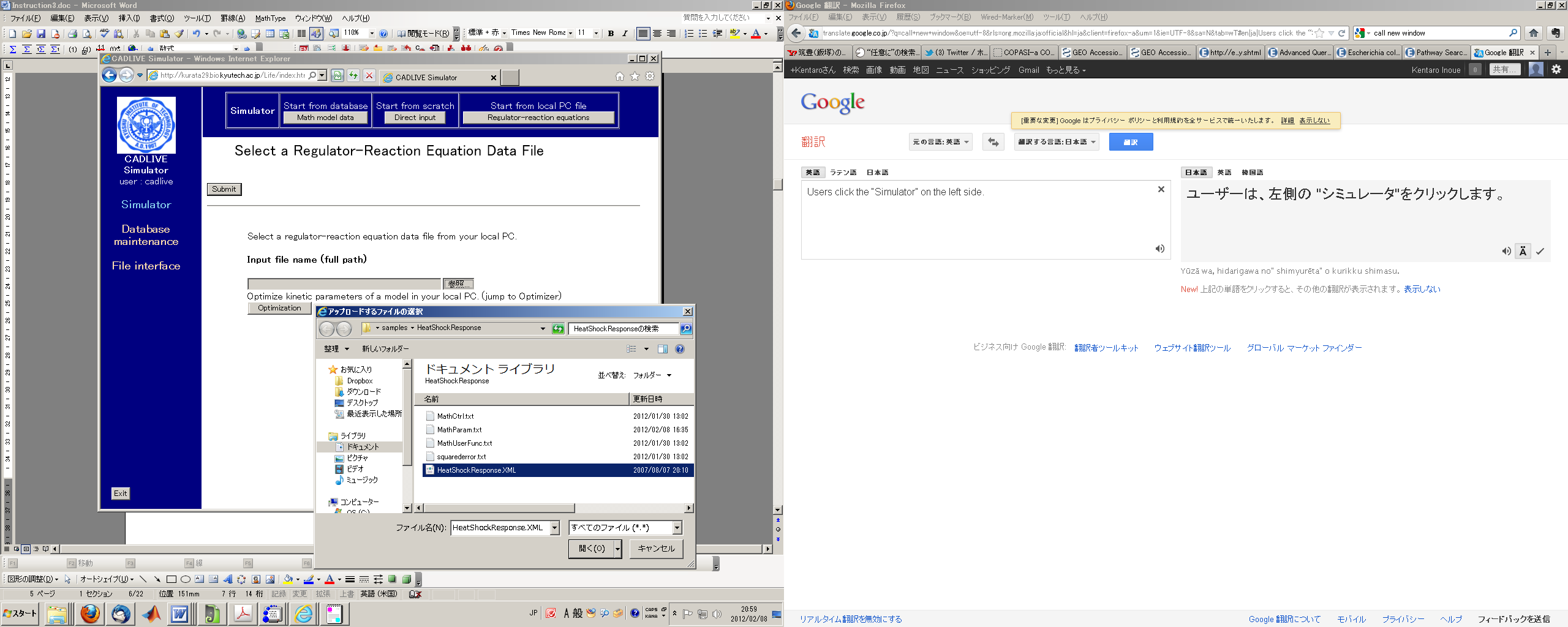


Fig.7 Upload of a CADLIVE format file

Users select a mathematical model. Here, select “TPP_RAPID”.


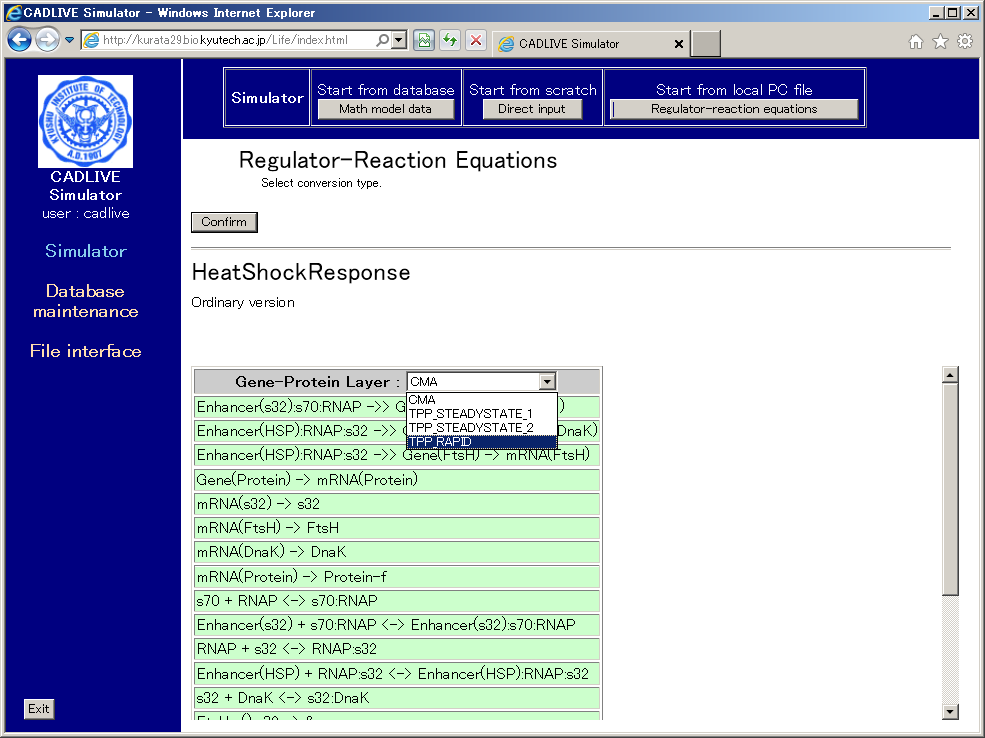


Fig.8 Selection of type of a mathematical model

Users can edit the mathematical equations.


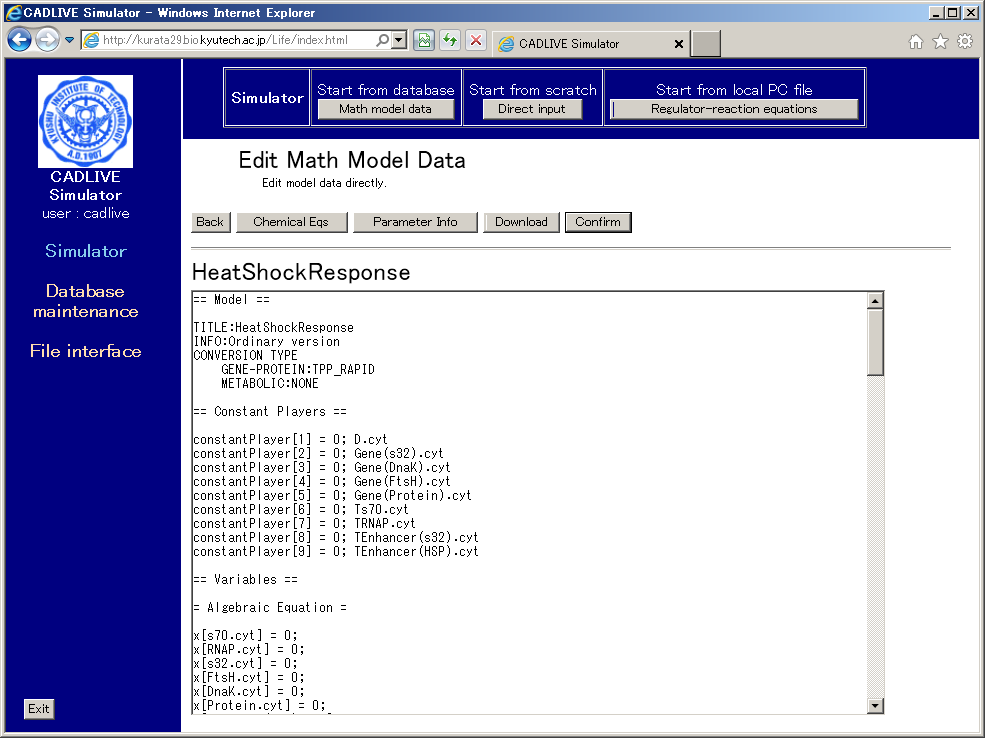


Fig.9 Mathematical equations

Users can select an analysis type. Here, select “Dynamic”.


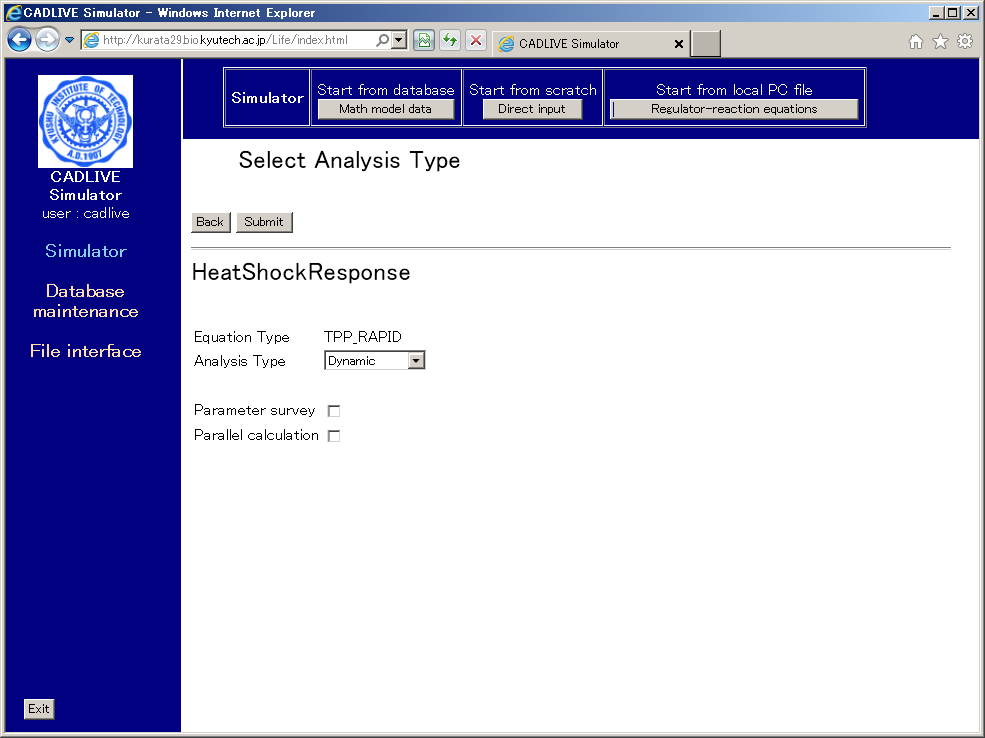


Fig.10 Selection of analysis type

Users set a control data for simulation. Here, select the adaptive Runge-Kutta as the solver type and set the end time to 100, time step-size 0.1, and monitoring interval 1. The others are set to the default values.


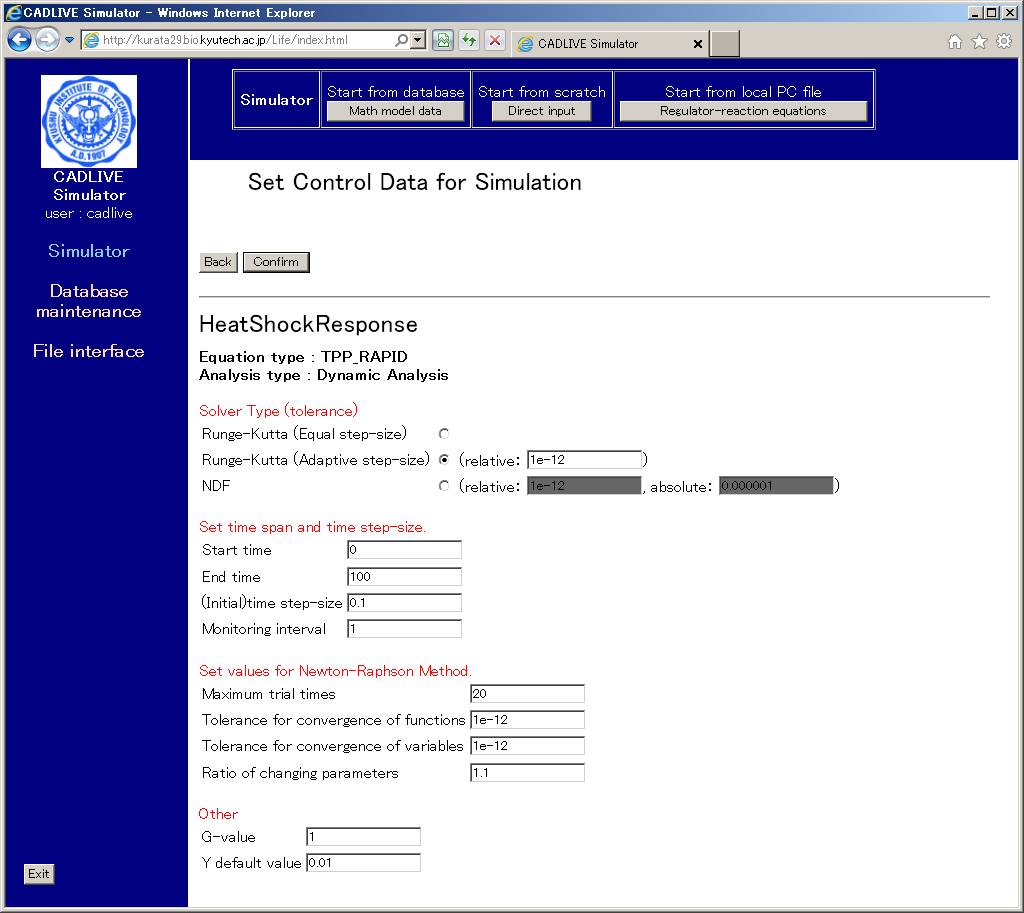


Fig.11 Setting of control data for simulation

Users provide the values of the kinetic parameters and initial concentrations to the mathematical equations and can set some events. Here, set the parameter values, initial values and events, which are written in the file “MathParam.txt” in the folder “HeatShockResponse” in **Additional file 3**. The event is a heat shock.


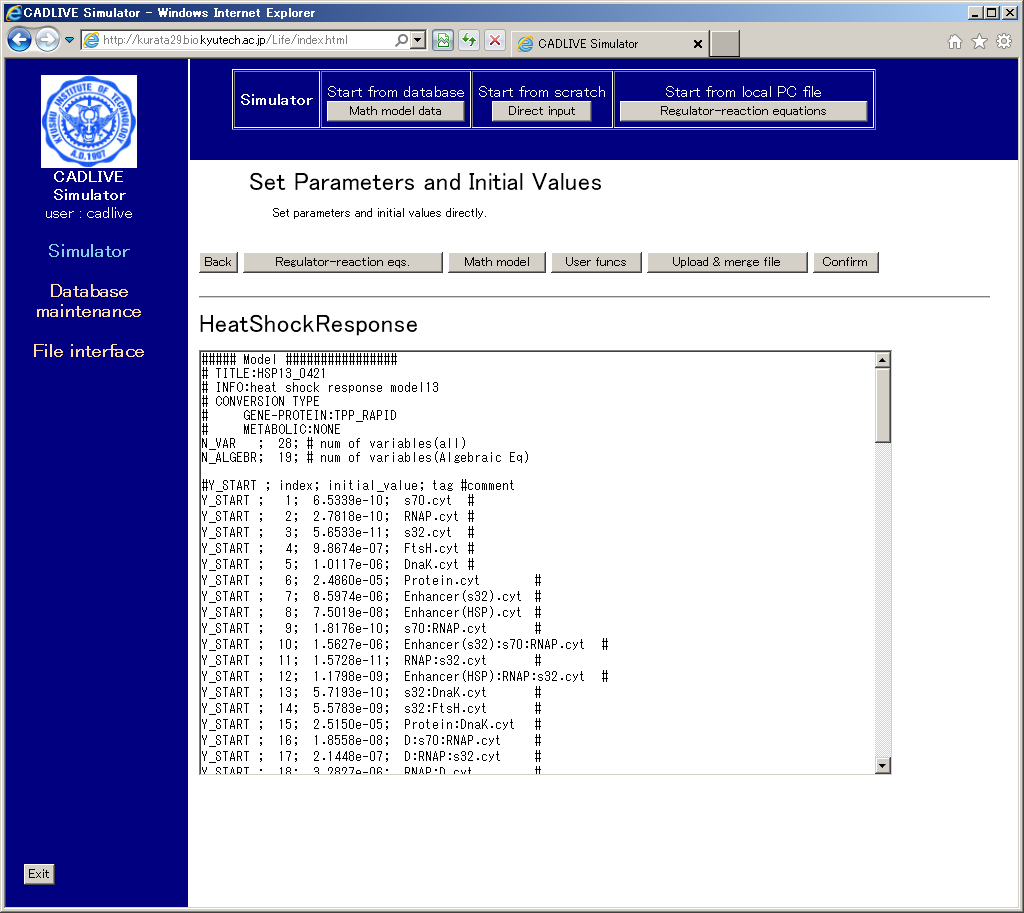


Fig.12 Setting of kinetic parameters and initial values

Users confirm the parameters and values and open the CADLIVE Optimizer.


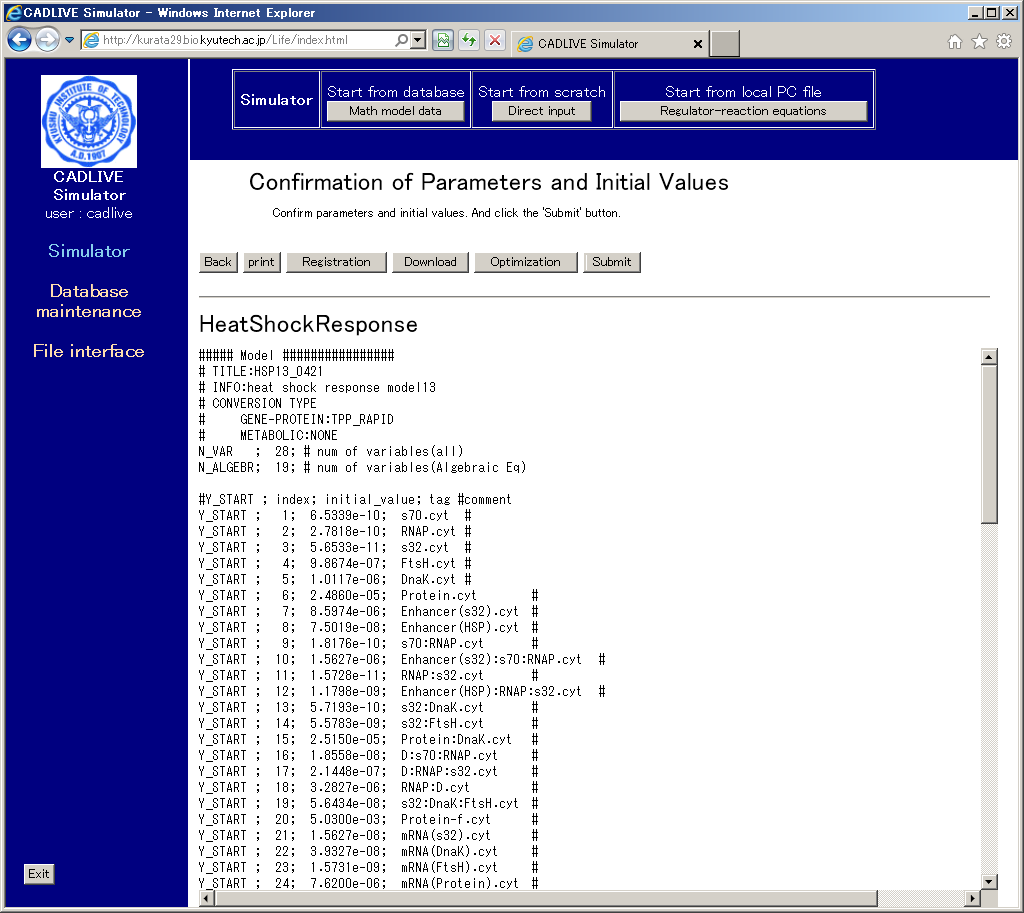


Fig.13 Confirmation of parameters and initial values and link to the CADLIVE Optimizer

# 4. Optimization of the kinetic parameters of the equations by the CADLIVE Optimizer

By clicking the “Optimizer” button in **Fig.7** or the “Optimization” button in **Fig.13**, the CADLIVE Optimizer is opened. The CADLIVE Optimizer selects either of the two approaches without and with mathematical conversion. If users download MathParam.txt, MathCtrl.txt, and MathUserFunc.txt from the CADLIVE Dynamic Simulator, users can start the CADLIVE Optimizer from **Fig.7** without any mathematical conversion. This case is applicable when users optimize the existing model. Usually, users start an optimization from **Fig.13**. Setting of the CADLIVE Optimizer has three steps: GA parameter setting, search parameter setting, and user function setting.

## 4-1. GA parameter setting

Users set the encode method, GA type, digenesis, immigration, crossover, and mutation for GA. First, users select either of the two ways to create a GA parameter set on the screen and to upload the existing GA parameter set file (**Fig.15**). Both ways can edit the parameters (**Fig.16**). The function of each parameter is described in **Table 1, 2**. Here, the maximum generation number is set to 30 and the others are set as the default values.


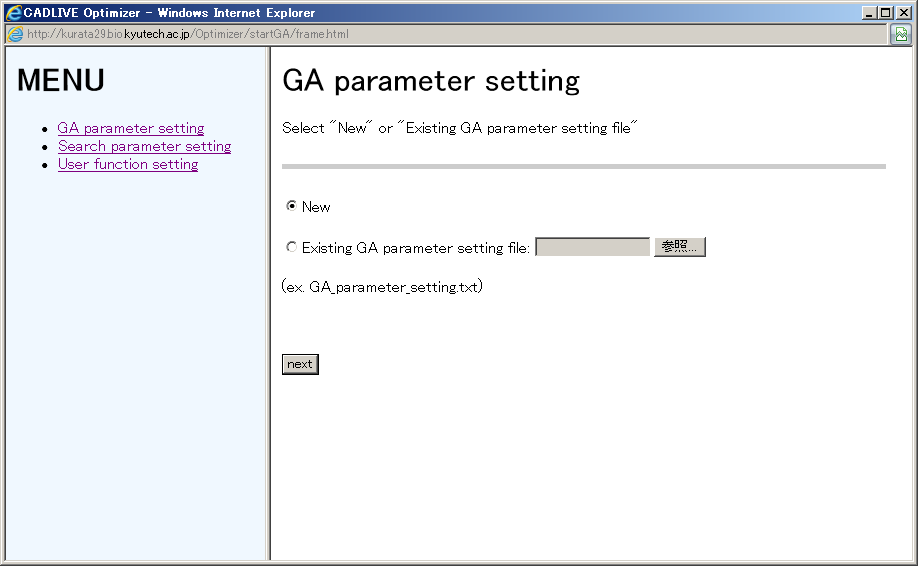


Fig.15 GA parameter setting


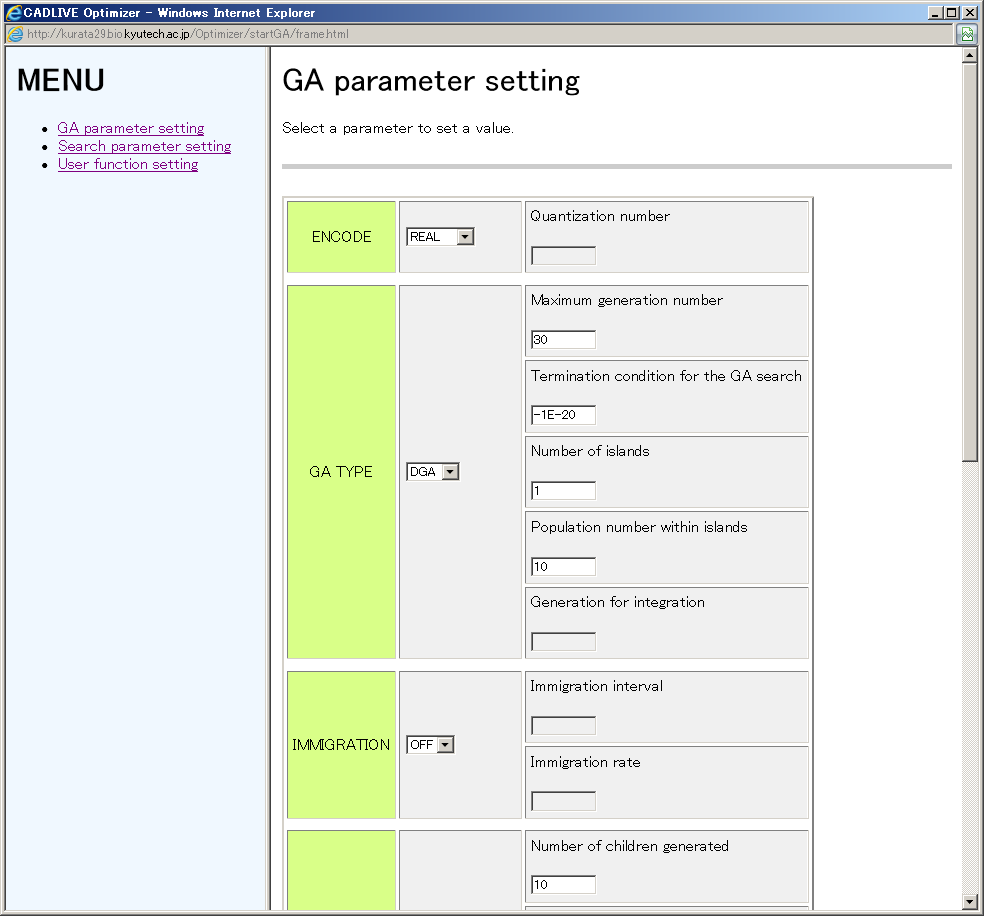


Fig.16 GA parameter setting screen

Table 1 Control parameters for setting GAs. Key words’ alternatives are selected. Both real-coded GA (RGA) and bit-string (BGA) can select the transparent alternatives. The thin gray alternatives can be selected only by RGA, and the dark gray alternatives can be selected only by BGA.

| Key words | Alternatives | Meanings |
| --- | --- | --- |
| ENCODE | REAL | Real GA |
| (Encode method) | BINARY | Binary coding bit string type GA |
|  | GRAY | Gray coding bit string type GA |
| GATYPE | DGA | Distributed GA (island model) |
| (Island model) | DIGA | Distributed and Integrated GA |
| IMMIGRATION | ON | Immigration |
|  | OFF | No immigration |
| DIGENESIS | NORMAL | Normal generation |
| (Generation alternation) | MGG | MGG |
| CROSSOVER | BLX | Blend crossover |
| (Crossover method) | UNDX | Unimodal Normal Distribution crossover |
|  | UNDXm | Multi-parental Unimodal Normal Distribution crossover |
|  | SPX | Symplex crossover |
|  | NPOINTS | n-point crossover (BGA) |
|  | NONE | No crossover |
| MUTATION | RegionUni | Uniform mutation within region |
| (Mutation method) | FixedUni | Uniform mutation with fixed width |
|  | FixedNormal | Normal mutation with fixed width |
|  | VariableUni | Uniform mutation with variable width |
|  | VariableNormal | Normal mutation with variable width |
|  | BitReverse | Bit reverse mutation |
|  | NONE | None |

Table 2 Values where users are allowed to set with respect to each parameter.

| Parameter | Values |
| --- | --- |
| Quantization number | Integer $\geq$ 1 |
| Maximum generation number | Integer $\geq$ 1 |
| Value for terminating a search | real value (double type) |
| Number of islands | Integer $\geq$ 1 |
| Population number within islands | variable number +2 $\leq$ Integer $\leq$ Maximum |
| Generation for integration | Integer $\geq$1 |
| Immigration interval | Integer $\geq$ 1 |
| Immigration rate | Real value [0,1] |
| Number of children generated by MGG | 1 $\leq$ Integer $\leq$ Maximum |
| Number of elites | Integer $\geq$ 0 |
| Selection rule | Roulette, Tournament, Random |
| Size of tournament | 1 $\leq$ Integer $\leq$(population number of islands – number of parents +1) |
| alpha | Real value > 0 |
| beta | Real value > 0 |
| M | 1 $\leq$ Integer $\leq$ number of variables |
| epsilon | Real value > 0 |
| N | Integer$\geq$ 1 |
| Mutation rate | Real value [0,1] |
| Parameter range | Real value > 0 |
| Standard deviation | Real value > 0 |

## 4-2. Search parameter setting

Users select the search (kinetic) parameters and can edit the initial values of all the parameters (**Fig.17**). Here, kx[1] and kp[3] are selected as the search parameters.


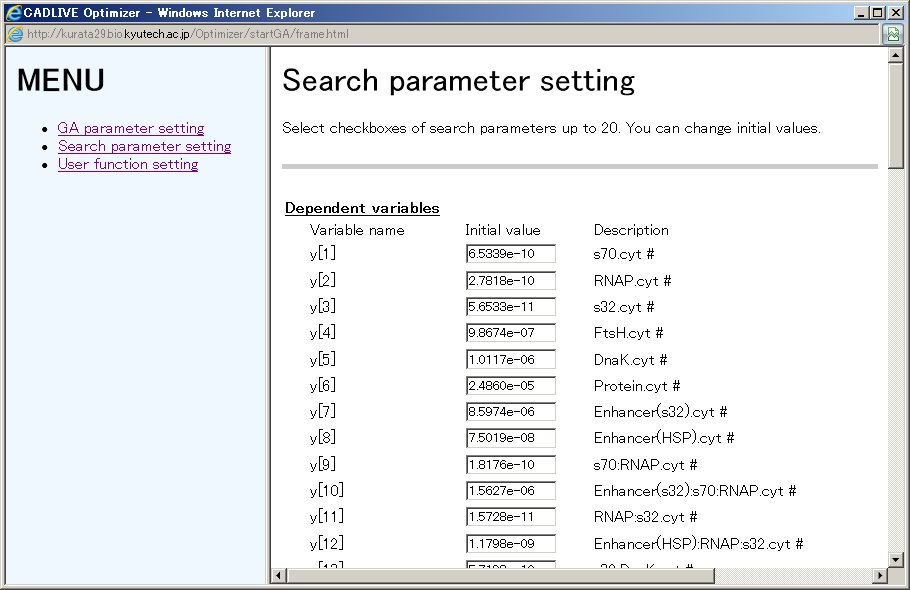


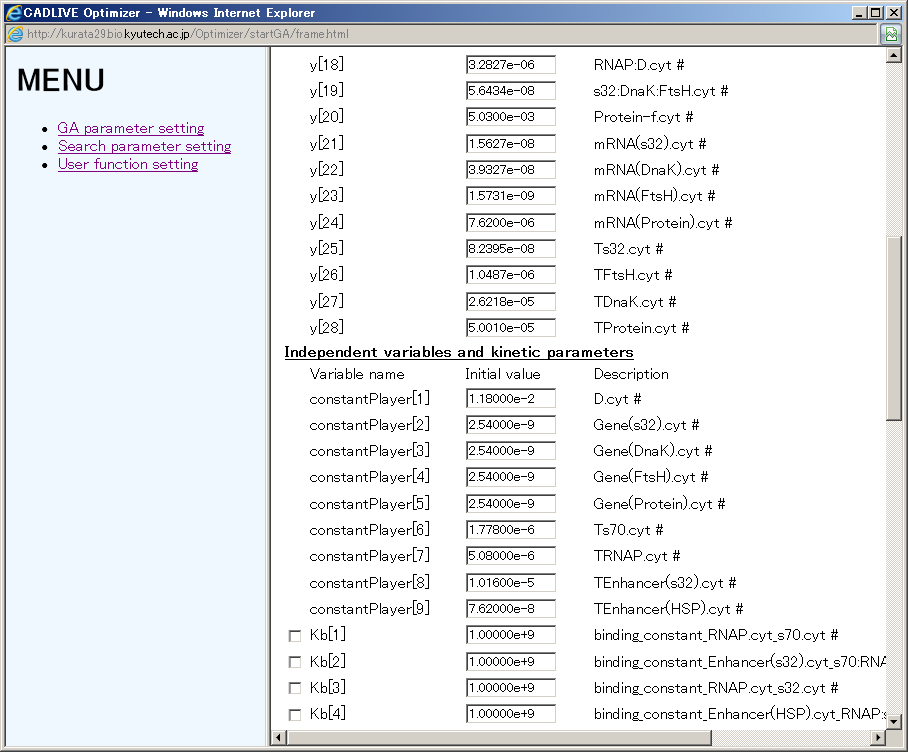


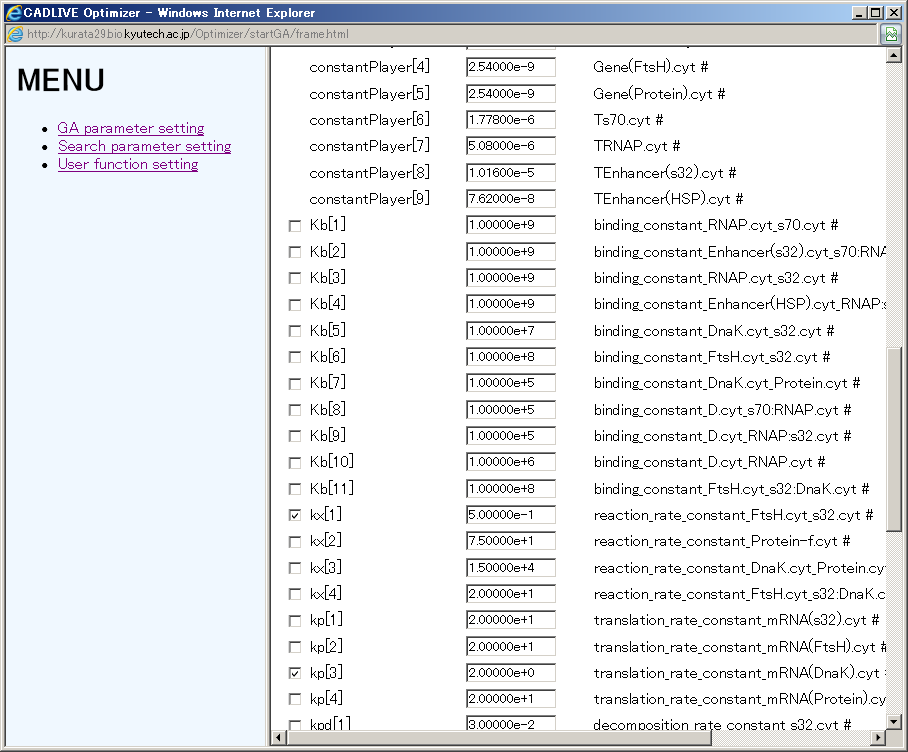


Fig.17 Selection of search parameters

Users set the search ranges (the minimum and maximum values) of the selected parameters (**Fig.18**). By default, the parameter search ranges are inputted $(\text{initial value})\times0.1$ and $(\text{initial value})\times10$ as the minimum and maximum values, respectively. Here, set them to the default values.


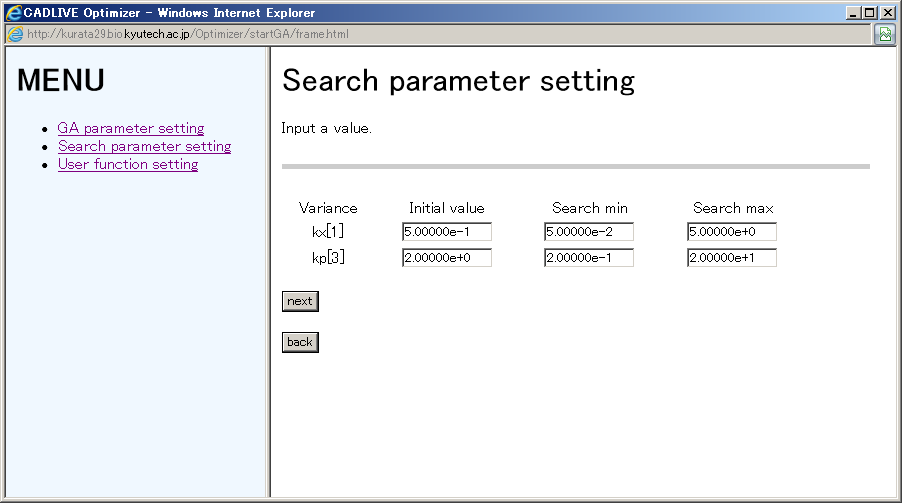


Fig.18 Parameter search range setting

## 4-3. User function setting

Users write an objective function below /*Input an objective function*/ (**Fig.19**) according to the C language. y[“row”][“col”] indicates the time-dependent variable, where “row” is the monitoring index and “col” is the dependent variable index. num_row is the number of monitoring and num_col is the number of dependent variables. ls_ret is the error code. $*fitness=0$ indicates that the parameter set is completely optimized with respect to the objective function. An index value of “col” > 0 in y corresponds to the index of Y_START in **Fig.12** or y in **Fig.17** and a value of “row” ≥0 corresponds to the index of time. In the heat shock response system, y[0][1] indicates the initial value of the “s70”. Note that the objective function is defined as the maximization problem of $*fitness\leq0$.


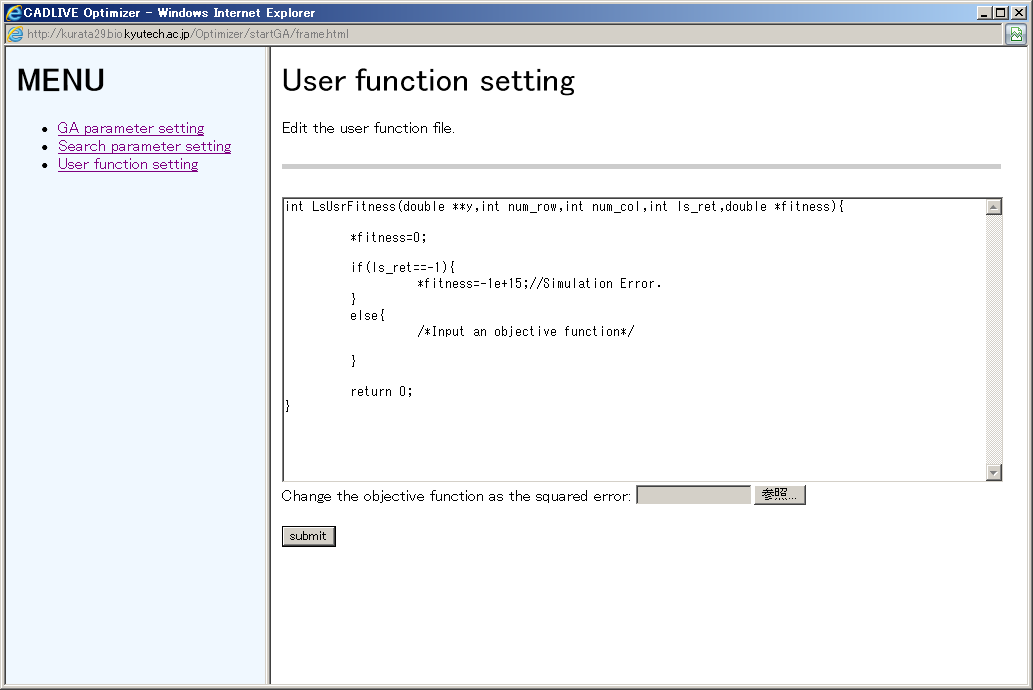


Fig.19 User function setting screen

Users can also set the objective function as the sum of squared errors (SSE). Users make a file with time-course data (**Fig.20**) such as experimental data. The objective function is set as the SSE when the file is uploaded. The SSE should be minimized for a parameter set *P*.

$SSE(P)=\sum_{i=1}^{N} \sum_{j=1}^{k} \left(\frac{x_{ij}(P)-y_{ij}}{y_{ij}}\right)^{2}$,

where *x_ij_*(*P*) are the simulated data corresponding to the experimental or reference data *y_ij_*. *N* is the number of molecules for optimization. *k* is the number of the experimental data.

Here, the objective function is defined as the SSE using the data (**Fig.20**) as the reference data.


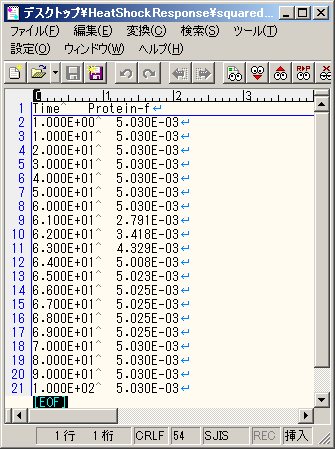


Fig.20 The reference time course data necessary for using the SSE as the objective function

## 4-4. GA execution

After confirming the user function setting, the optimization process starts by clicking the “startGA” button. If **Fig.21** is displayed, it indicates that the optimization programs are successfully compiled and being executed. The progress of the search by GAs is displayed when clicking the “progress” button.


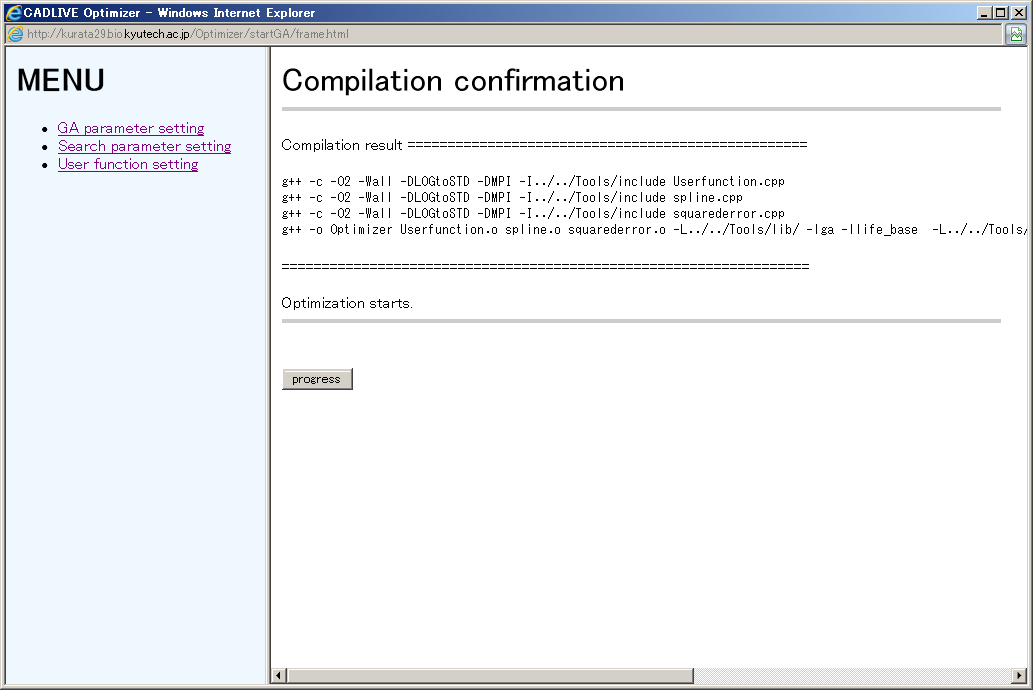


Fig.21 Compilation confirmation screen

During the optimization, **Fig.22** is displayed. The percentage indicates the progress status of the calculation that indicates the ratio of the calculated generation number to the maximum generation number. By clicking the “reload” button, the percentage of the current progress status is displayed on the screen. If it takes a long time (the percentage does not change in a little while), users should register users’ e-mail address. In this case, the CADLIVE sever will send e-mail to let users know the completion of the optimization. Users can forcibly stop the calculation by clicking the “stop” button. When the calculation time needs more than one week, the calculation should be forcibly stopped and the server will notice to users that information.


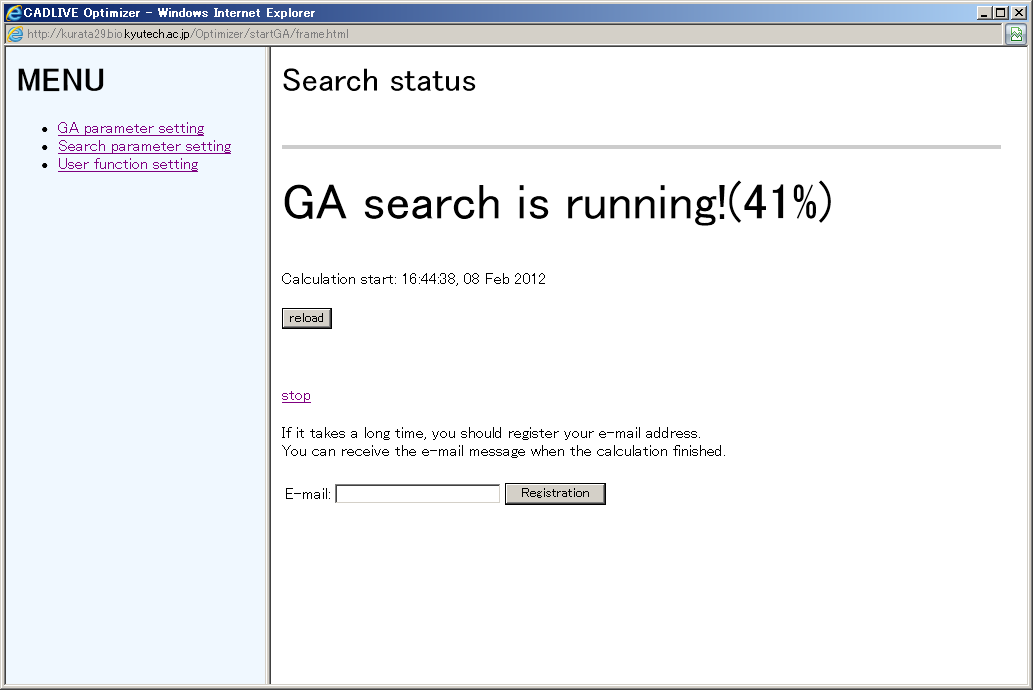


Fig.22 GA running screen

## 4-5. Confirmation of the results

When the calculation is finished, **Fig.23** is displayed. Users can gain three results (**Fig.24**): Simulation, Download, and Process of fitness.


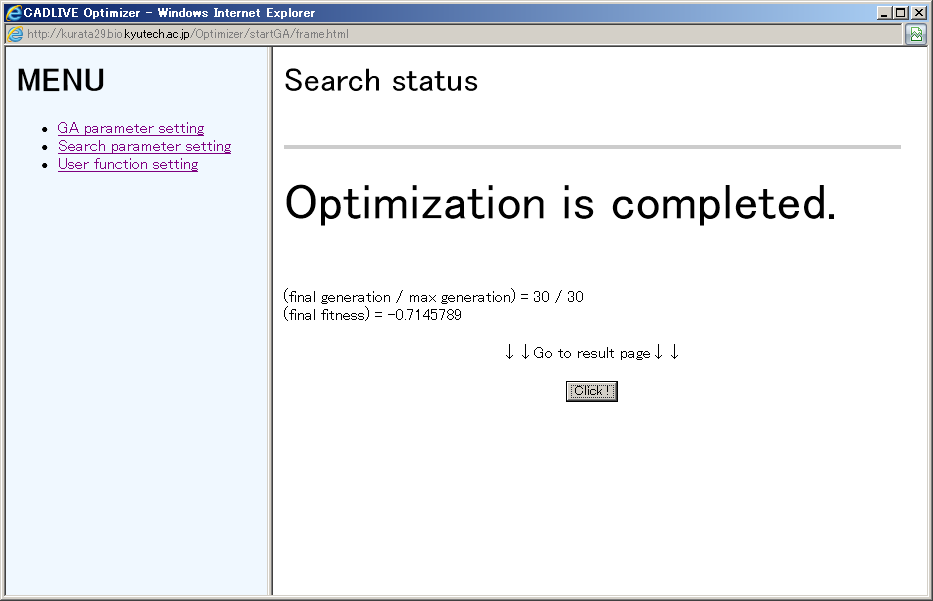


Fig.23 Calculation finish screen


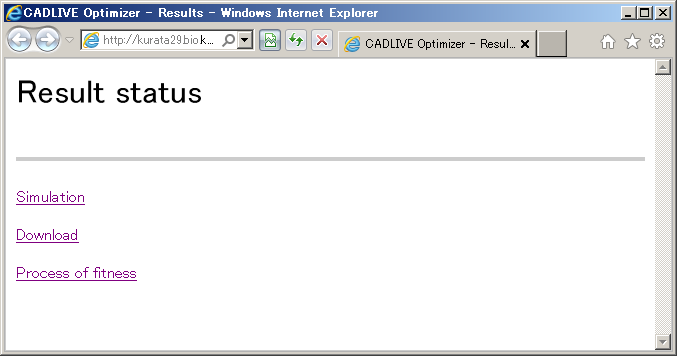


Fig.24 Links of the three results

### 4-5-1. Simulation

By clicking the “Simulation” button in **Fig.24**, users can simulate the mathematical model with the optimized parameters. The folded protein shows a peak after the heat shock (**Fig.25**).


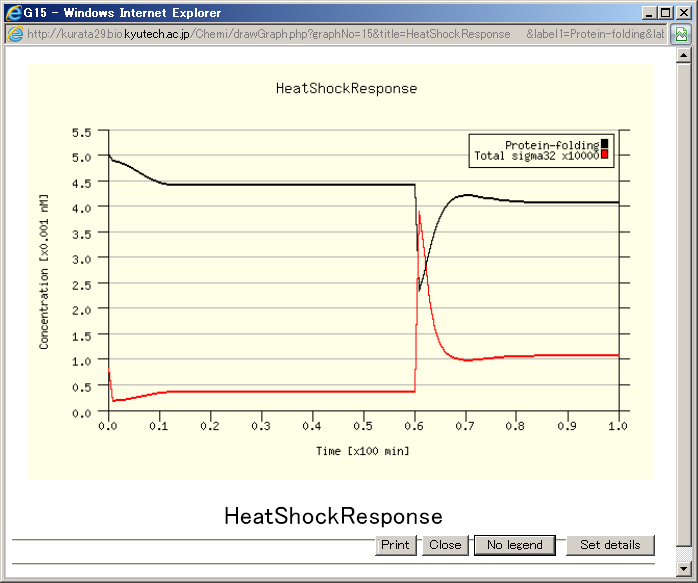


Fig.25 Simulation result

### 4-5-2. Download the result files

By clicking the “Download” button in **Fig.24**, users can download the following five files (**Fig.26**).

Three input files:

GA parameter setting file, Search parameter setting file, and User function setting file.

Two output files:

GA result file that stores the fitness and the values of the search parameters of the entire individuals in each island every generation, and

Optimized search parameter file that stores the optimized parameter values.


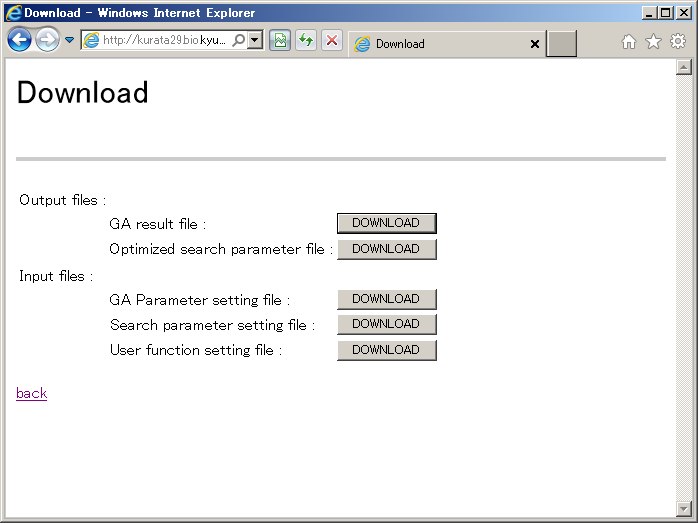


Fig.26 Download screen

### 4-5-3. Process of fitness

By clicking the “Process of fitness” button in **Fig.24**, the change in the fitness value with respect to generation is displayed (**Fig.27**).


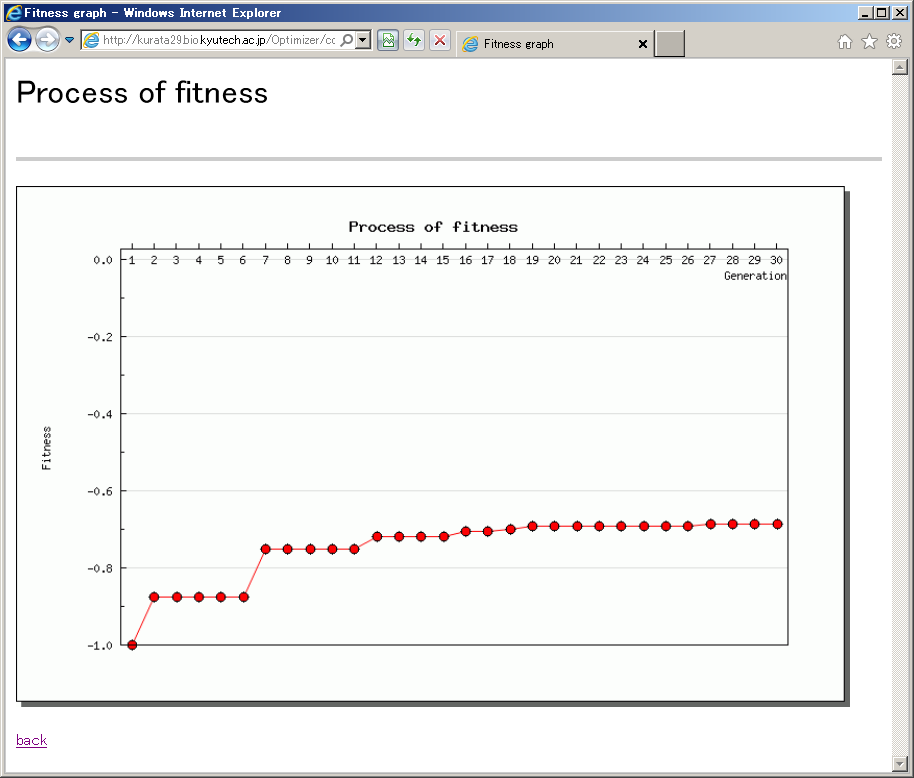


Fig.27　Process of fitness

# 5. References

1. Kurata H, Matoba N, Shimizu N (2003) CADLIVE for constructing a large-scale biochemical network based on a simulation-directed notation and its application to yeast cell cycle. Nucleic Acids Res. 31: 4071-4084.

2. Kurata H, Inoue K, Maeda K, Masaki K, Shimokawa Y, et al. (2007) Extended CADLIVE: a novel graphical notation for design of biochemical network maps and computational pathway analysis. Nucleic Acids Res. 35: e134.

3. Kurata H, Masaki K, Sumida Y, Iwasaki R (2005) CADLIVE dynamic simulator: direct link of biochemical networks to dynamic models. Genome Res. 15: 590-600.
