# Supplementary material for: CADLIVE optimizer: web-based parameter estimation for dynamic models
Source: Source Code Biol Med. 2012 Aug 28;7:9. doi: 10.1186/1751-0473-7-9 (PMC3457844; doi:10.1186/1751-0473-7-9)
Supplement: Additional file 3 — Description of three example models. [file 1751-0473-7-9-S3.doc]

**Additional file 3. Description of three example models**

# Simple enzyme reaction model

The enzyme reaction model consists of a substrate, a product, and an enzyme. The enzyme catalyzes the conversion of the substrate to the product. The biochemical network is constructed by the CADLIVE GUI Network Constructor, shown in **Fig.1**. Then, the CADLIVE Dynamic Simulator automatically converts the biochemical network into the mathematical equations. Here, we employ the CADLIVE Optimizer to estimate the kinetic parameters with an objective function. In GA parameter setting, the number of maximum generation, which is the number of maximum iterations of the calculation, is 50, the islands are 10, the populations with the each island are 10, crossover algorithm is UNDX, and generation alternation is MGG. The objective (fitness) function is defined by:

$fitness=-\left(\frac{y(1)_{steady-state}-10^{-3}}{10^{-3}}\right)^{2}$,

where *y*(1) is the concentration of the product and its steady state concentration is adjusted to a value of 10^-3^. The result shows the simulation with the objective function (**Fig.2**).


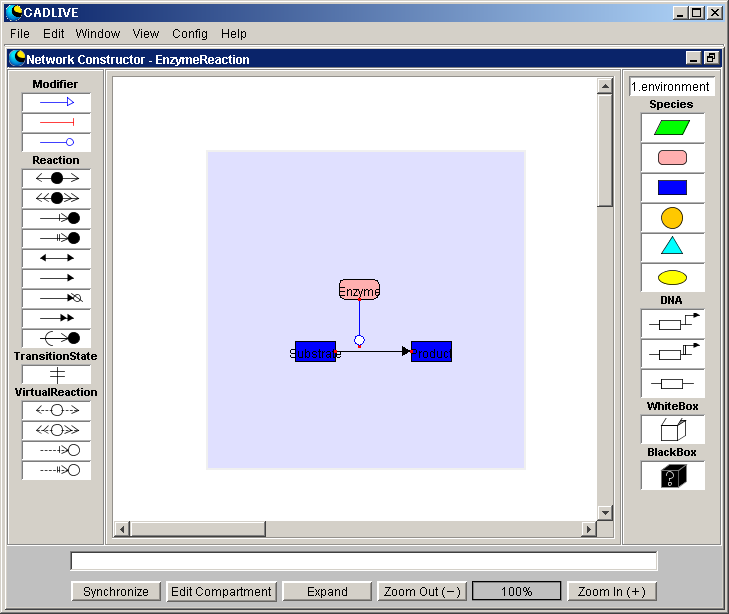


Fig.1 Network map of the simple enzyme reaction drawn by the CADLIVE GUI Network Editor


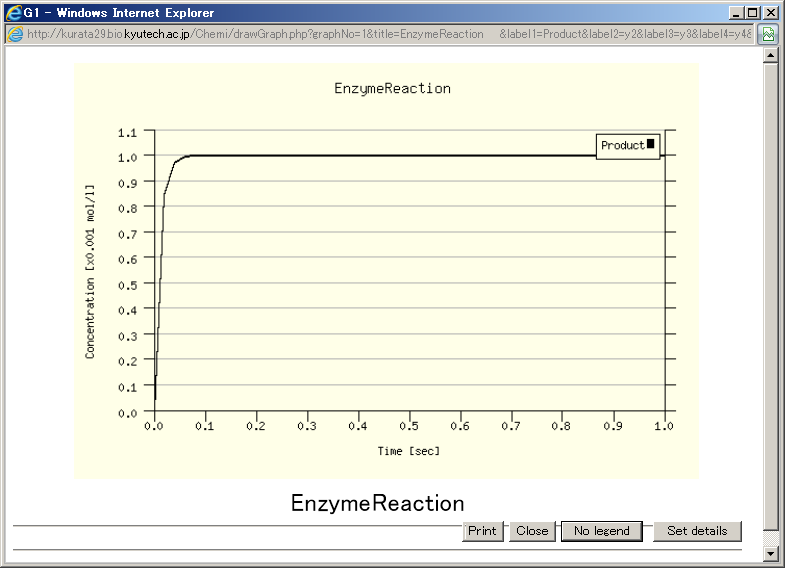


Fig.2 Simulation of the optimized simple enzyme reaction

# Heat shock response system

Heat shock causes unfolding, misfolding, or aggregation of proteins at the cellular level on the order of seconds, compromising cellular function. Cells overcome the heat stress by initiating the production of heat-shock proteins that act as chaperones to refold denatured proteins into their native states, or proteases that degrade them. In this paper, we use the previous model made by the CADLIVE GUI Network Constructor (**Fig.3**) and the model is converted into mathematical equations by the CADLIVE Dynamic Simulator. In this simulation, heat shock occurs at 60 min by changing the rate constant for denaturing proteins and translation for σ^32^. In that time, the normal folded protein is reproduced to recover after the heat shock. In GA parameter setting, the number of maximum generation is 30, the islands 1, the populations with the each island 10, crossover algorithm UNDX, and generation alternation MGG. The objective function is defined as the SSE.

$fitness=-\sum_{i=1}^{k} \left(\frac{x_{i}(20)-y_{i}(20)}{y_{i}(20)}\right)^{2}$,

where *x_i_*(20) and *y_i_*(20) is the simulated and experimental folded protein concentrations respectively. *k* is the number of experimental data. The simulated concentration on time *i* is interpolated using the spline interpolation function. This experimental data indicate that the folded protein recovers into the normal state again after the heat shock. The result shows that the folded proteins recover after the heat shock compared with before the optimization (**Fig.4**).


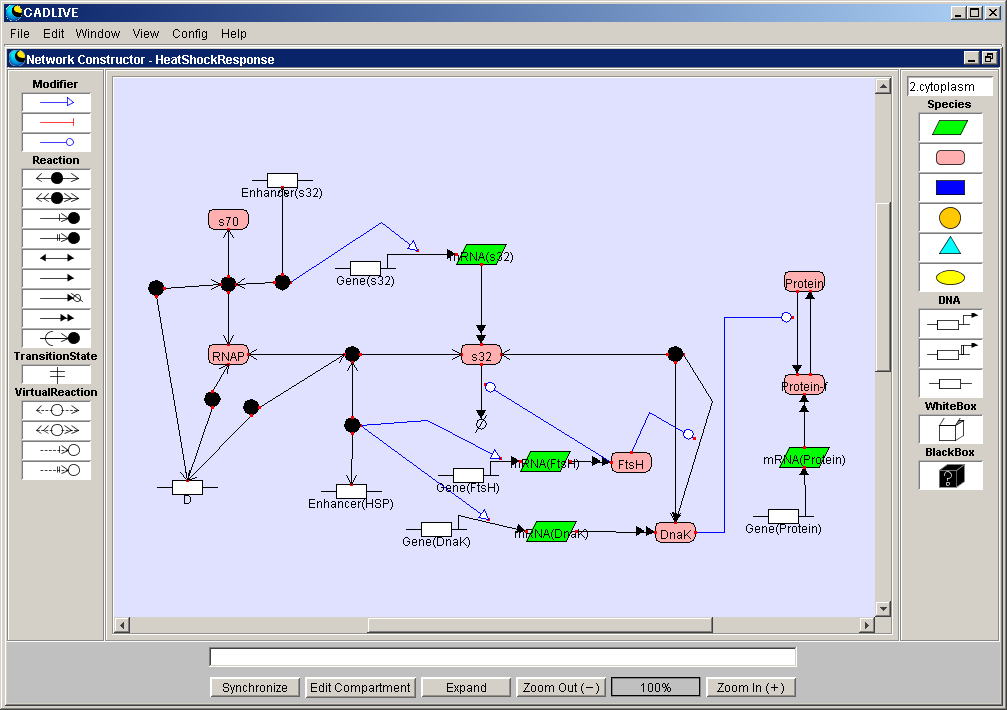


Fig.3 Network map of the heat shock response system drawn by the CADLIVE GUI Network Editor


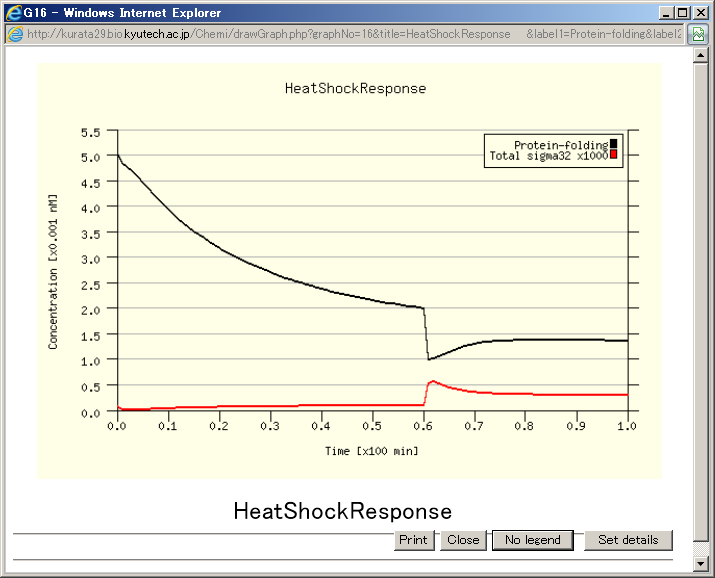


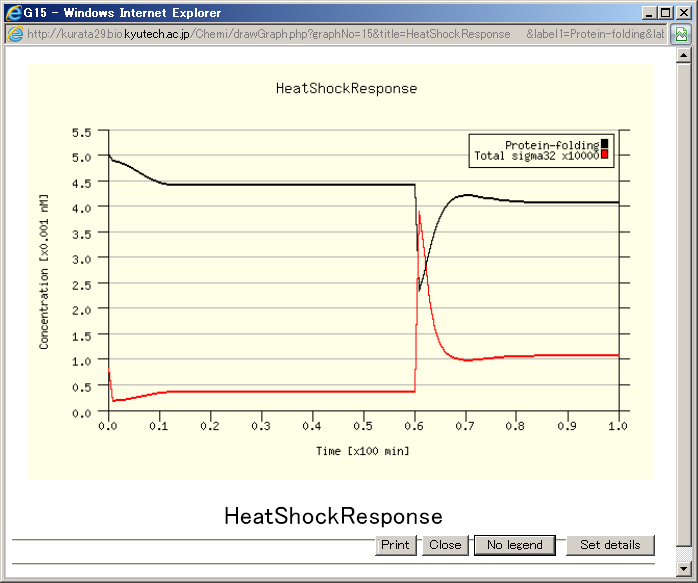


Fig.4 Simulation of the heat shock response system before (top) and after (bottom) optimization.

# Nitrogen assimilation system

In nitrogen assimilation system, glutamine and glutamate are synthesized by adding ammonia to 2-ketoglutarate by glutamine synthetase (GS), glutamate synthase (GOGAT), and glutamate dehydrogenase (GDH). In a low concentration of ammonia, GS plays a major role in the ammonia assimilation. This system has multiple feedbacks that provide a robust property for the intracellular N/C ratio of the concentration of the nitrogen source (N) to that of the carbon source (C) against environmental stress. Here, we use the previous model made by the CADLIVE GUI Network Constructor (**Fig.5**) and the model is converted into the mathematical equations by the CADLIVE Dynamic Simulator. In this simulation the ammonia concentration decreased to one fifth on 1000 min. In GA parameter setting, the number of maximum generation is 30, the islands 1, the populations with the each island 10, crossover algorithm UNDX, and generation alternation MGG. Assuming that the nitrogen assimilation system is able to maintain the ratio of glutamine (nitrogen source) to 2-ketoglutarate (carbon source) against the remarkable change in the ammonia concentration, the objective function is defined by:

$fitness=-\left|1-\frac{y(46)/y(47)|_{low\text{ }ammonia\text{ }concentration}}{y(46)/y(47)|_{high\text{ }ammonia\text{ }concentration}}\right|$,

where y(46)/y(47) is the ratio of the glutamine concentration to the 2-ketoglutarate concentration at the steady state. The optimized model shows that the N/C ratio recovers after the decrease in the ammonia concentration (**Fig.6**).


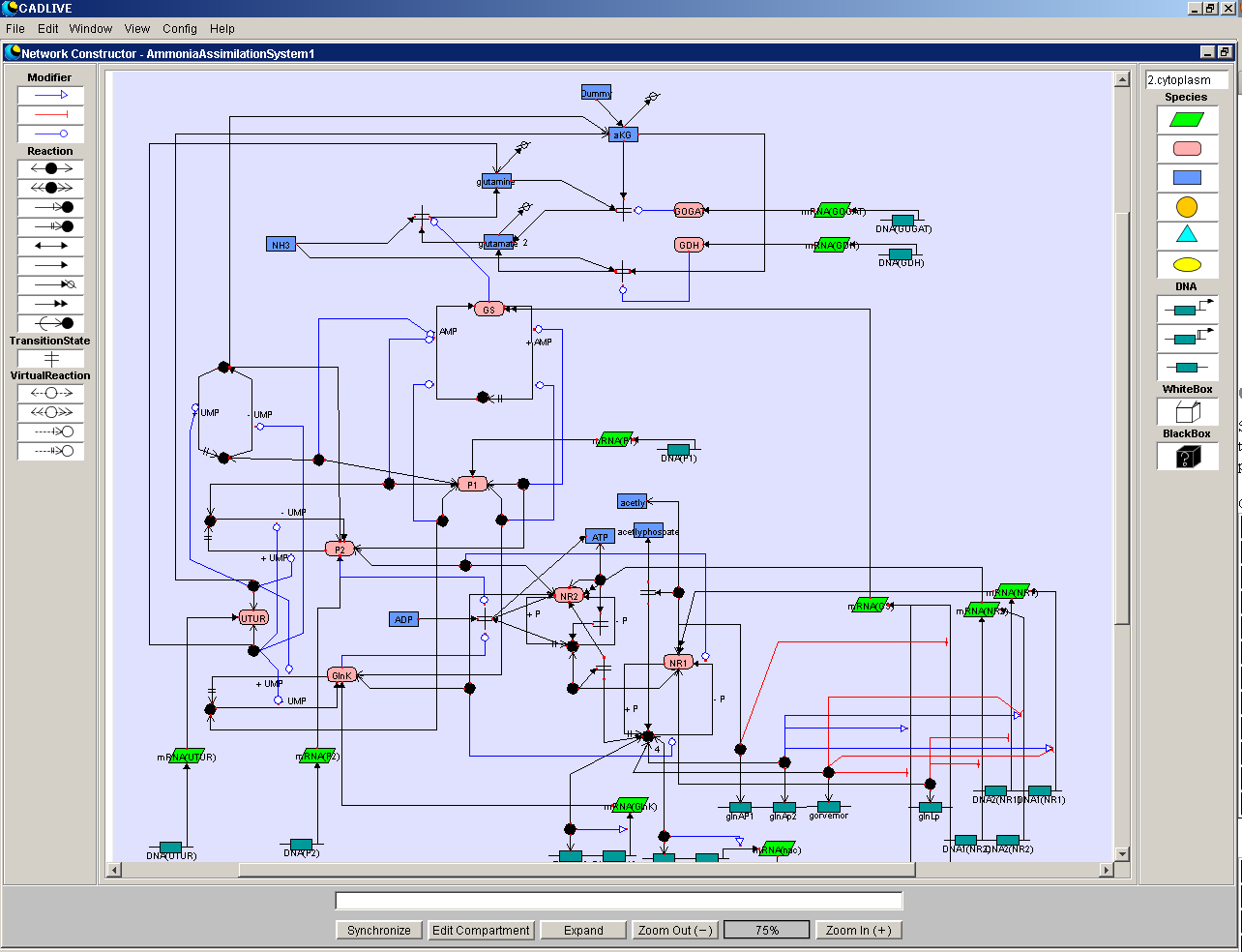


Fig.5 Network map of the nitrogen assimilation system drawn by the CADLIVE GUI Network Constructor.


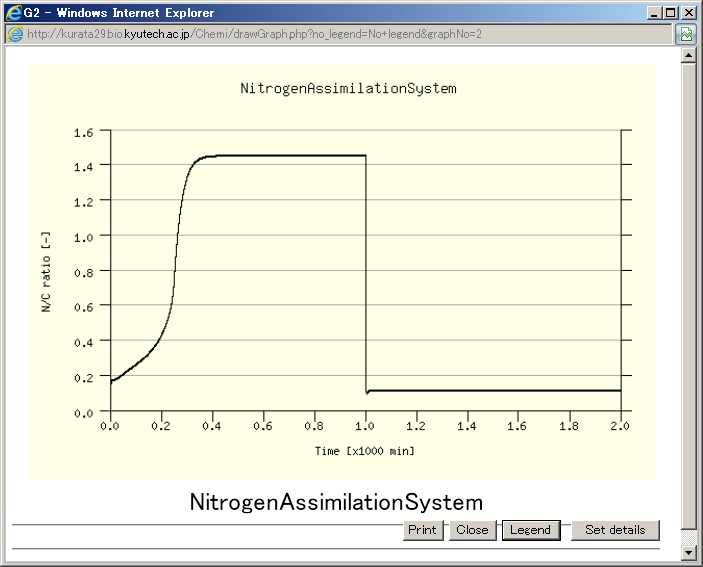


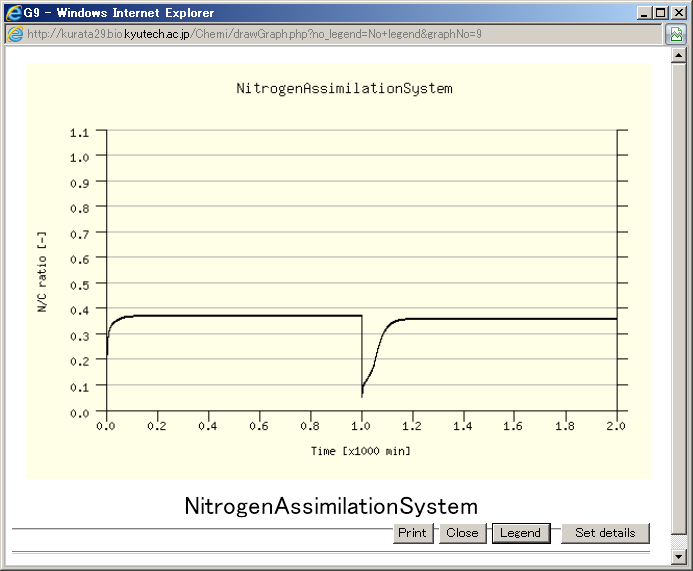


Fig.6 Simulation of the nitrogen assimilation system before (top) and after (bottom) optimization.

References

[1] H. Kurata, H. El-Samad, R. Iwasaki, H. Ohtake, J.C. Doyle, I. Grigorova, C.A. Gross, M. Khammash, Module-based analysis of robustness tradeoffs in the heat shock response system, PLoS Comput. Biol., 2 (2006) e59.

[2] K. Maeda, H. Kurata, Quasi-multiparameter sensitivity measure for robustness analysis of complex biochemical networks, J Theor. Biol., 272 (2011) 174-186.

[3] H. Kurata, K. Masaki, Y. Sumida, R. Iwasaki, CADLIVE dynamic simulator: direct link of biochemical networks to dynamic models, Genome Res., 15 (2005) 590-600.

[4] K. Masaki, K. Maeda, H. Kurata, Biological design principles of complex feedback modules in the E. coli ammonia assimilation system, Artificial life, 18 (2012) 53-90.
